# Quinoa Secondary Metabolites and Their Biological Activities or Functions

**DOI:** 10.3390/molecules24132512

**Published:** 2019-07-09

**Authors:** Minyi Lin, Peipei Han, Yuying Li, Weixuan Wang, Daowan Lai, Ligang Zhou

**Affiliations:** Department of Plant Pathology, College of Plant Protection, China Agricultural University, Beijing 100193, China

**Keywords:** quinoa (*Chenopodium quinoa*), secondary metabolites, biological activities, functions

## Abstract

Quinoa (*Chenopodium quinoa* Willd.) was known as the “golden grain” by the native Andean people in South America, and has been a source of valuable food over thousands of years. It can produce a variety of secondary metabolites with broad spectra of bioactivities. At least 193 secondary metabolites from quinoa have been identified in the past 40 years. They mainly include phenolic acids, flavonoids, terpenoids, steroids, and nitrogen-containing compounds. These metabolites exhibit many physiological functions, such as insecticidal, molluscicidal and antimicrobial activities, as well as various kinds of biological activities such as antioxidant, cytotoxic, anti-diabetic and anti-inflammatory properties. This review focuses on our knowledge of the structures, biological activities and functions of quinoa secondary metabolites. Biosynthesis, development and utilization of the secondary metabolites especially from quinoa bran were prospected.

## 1. Introduction

Quinoa (*Chenopodium quinoa* Willd.), a dicotyledonous plant belonging to Chenopodiaceae family, is one of the oldest native crops in the Andean region of South America, with approximately 7000 years of cultivation [1]. It has been considered as a pseudo-cereal because of the grain characteristics [2]. Consumption of seeds is the most common use of quinoa. Once the bran (also called hull or seed coats) containing saponins has been eliminated, the seeds can be consumed as entire grains or milled to flour for preparation of bread and pastry. The other parts such as leaves and stems were used as feed [2,3].

Quinoa has been recognized as a complete food due to a variety of vitamins, significant amounts of minerals, unsaturated fatty acids, dietary fiber, abounding proteins, and excellent balance of essential amino acids. The year 2013 was named “The Internaitonal Year of Quinoa” by the UN. Quinoa has been introduced and cultivated all over the world in the past ten years [3,4,5,6,7,8].

Quinoa possesses a large number of secondary metabolites, such as phenolic acids, flavonoids, terpenoids, steroids, and nitrogen-containing compounds. These metabolites play various physiological and ecological roles against harmful microorganisms, birds and insects. They also exhibit features beneficial to humans, including anti-diabetic [9], anticancer [10], cytotoxic [11], antimicrobial [12], anti-inflammatory [13], immunoregulatory [14] and adjuvant activities [15].

To our knowledge, there are many reviews on quinoa, most of them are focused on the nutritional, functional and antinutritional aspects [16,17,18], abiotic stress responses [19], biodiversity and sustainability [20], or only a specific topic of quinoa secondary metabolites and their biological activities such as steroids [21,22] and triterpenoid saponins [23], but no review covers almost all secondary metabolites and their biological activities. In this review, we summarize and discuss quinoa secondary metabolites on their structural diversity, biological activities or functions during the past 40 years.

## 2. Phenolic Acids and Their Biological Activities or Functions

About 29 phenolic acid analogues have been identified in quinoa. According to their structural features, they can be classified as benzoic acid analogues (**1**–**16**) and cinnamic acid analogues (**17**–**29**). Benzoic acid (**1**) was derived from cinnamic acid (**19**) *in planta* in the biosynthetic pathway of phenolic acids [24]. Phenolic acid derivatives are present in either free or conjugated forms. The total of conjugated phenolic acids in quinoa were at comparable level as that of free ones, suggesting that conventional solvent extraction and chromatographic analysis of extractable phenolic acids might have significantly underestimated the total phenolic acid content in quinoa, as such methods only detect free phenolic acids [25].

Phenolic acids can be released by acid, alkaline, and enzymatic treatments from the conjugated forms. It was reported that at least 19 phenolic acids were released in the residue of quinoa which can enhance bioaccessibility [25]. Bound phenolic acid derivatives in conjugated forms were not affected by environmental stresses [26]. Higher content of phenolic acids showed stronger antioxidant and inhibitory activities of α-glucosidase and pancreatic lipase [25].

### 2.1. Benzoic Acid Analogues and Their Biological Activities or Functions

**Figure 1:**
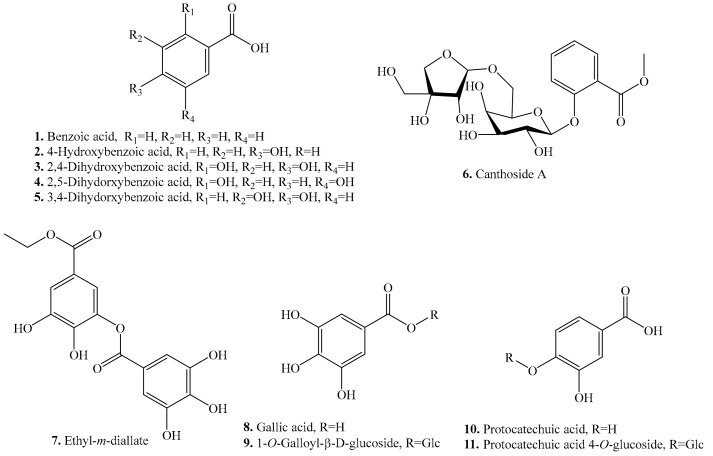

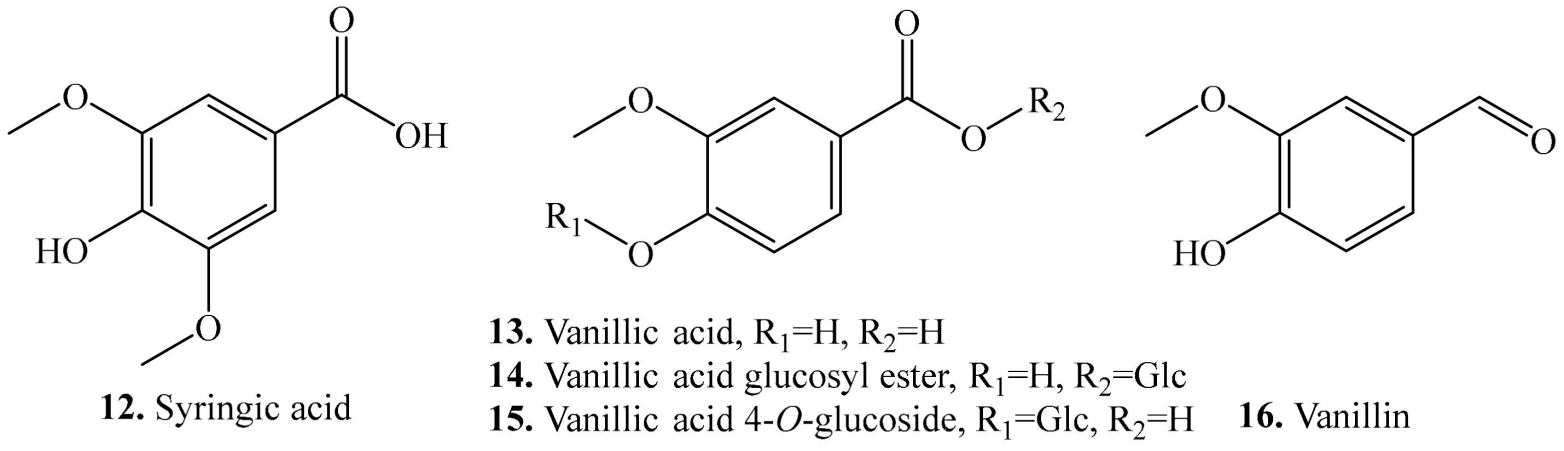
Structures of the benzoic acid analogues isolated from quinoa.

At least 16 benzoic acid analogues have been identified from quinoa. Their biological activities are listed in Table 1, and the structures are shown in Figure 1. Benzoic acid derivatives include benzoic acid (**1**), gallic acid (**8**), protocatechuic acid (**10**), syringic acid (**12**), vanillic acid (**13**), and their analogues. They are rich in the leaves and seeds of quinoa [25,27]. Though the benzoic acid analogues from quinoa have not been evaluated for their biological activities, these metabolites from other plant species have been reported to have antimicrobial [28,29], allelopathic [30], antioxidant [31], and antifeedant [32] activities (Table 1).

### 2.2. Cinnamic Acid Analogues and Their Biological Activities or Functions

**Figure 2:**
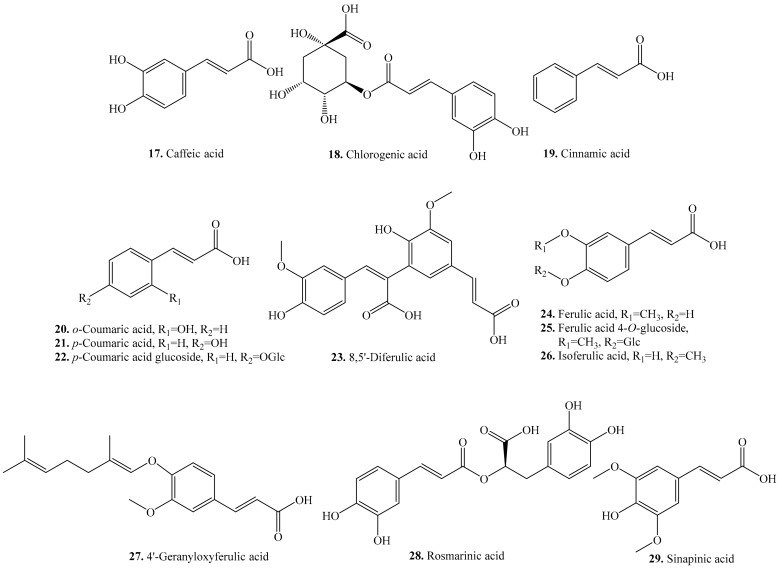
Structures of the cinnamic acid analogues isolated from quinoa.

Thirteen cinnamic acid analogues have been identified from quinoa. Their biological activities are listed in Table 2, and the structures are shown in Figure 2. These cinnamic acid derivatives include caffeic acid (**17**), chlorogenic acid (**18**), cinnamic acid (**19**), coumaric acid (**20**/**21**), ferulic acid (**24**), rosmarinic acid (**28**), sinapinic acid (**29**), and their analogues. Ferulic acid (**24**) and its derivatives were the predominant phenolics in bound form to be present in quinoa seeds [17].

Both ferulic acid (**24**) and sinapic acid (**29**) had more phytotoxic effects on cucumber seedling as compared to the other tested phenolic acids [54]. The phenolic acids from quinoa were also isolated from other plant species which showed a variety of biological activities such as antimicrobial [28], allelopathic [30], antioxidant [31], anti-apoptotic [55], anti-diabetic [56] activities that are mentioned in Table 2.

## 3. Flavonoids and Their Biological Activities or Functions

Flavonoids are based upon a fifteen-carbon skeleton consisting of two benzene rings linked via a heterocyclic pyrene ring [80]. They contain aglycones and their glycosides. The main flavonoid aglycones are kaempferol (**35**) and quercetin (**46**). Other aglycones in quinoa include acacetin (**30**), myricetin (**45**), daidzein (**62**), and genistein (**63**). According to the structural features, quinoa flavonoids can be classified as flavones (**30**–**33**), flavonols (**34**–**54**), flavanones (or dihydroflavones, **55**–**57**), flavanols (**58**–**60**), and isoflavones (**61**–**65**). Flavonoids play important roles in plants against the feeding insects and herbivores [81]. Flavonoids also have deterrent effects with respect to feeding and physiological behavior against some soil herbivorous nematodes [82].

### 3.1. Flavones and Their Biological Activities or Functions

**Figure 3:**
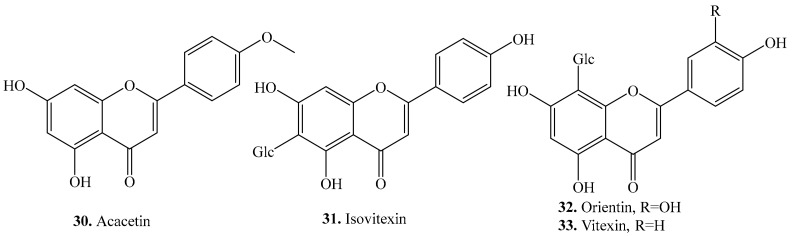
Structures of the flavones isolated from quinoa.

Four flavones, namely acacetin (**30**), isovitexin (**31**), orientin (**32**) and vitexin (**33**), have been identified from quinoa. Their biological activities are listed in Table 3, and the structures are shown in Figure 3. Flavones were significantly richer in sprouts than in other parts of quinoa. Quinoa sprouts grown in the darkness contained vitexin (**33**) and substantial amounts of isovitexin (**31**), whereas those grown in daylight only contained isovitexin (**31**). It is remarkable that no isovitexin (**31**) was present in quinoa seeds [34]. Acacetin (**30**), isovitexin (**31**), orientin (**32**) and vitexin (**33**) were also isolated from other plant species which showed various biological activities such as antioxidant [83], anti-inflammatory [84] activities, which are listed in Table 3.

### 3.2. Flavonols and Their Biological Activities or Functions

**Figure 4:**
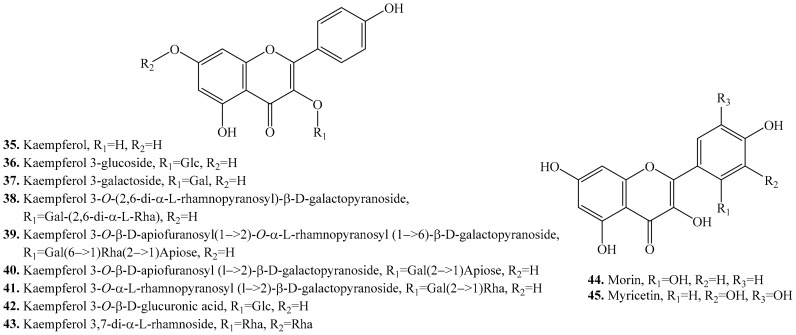

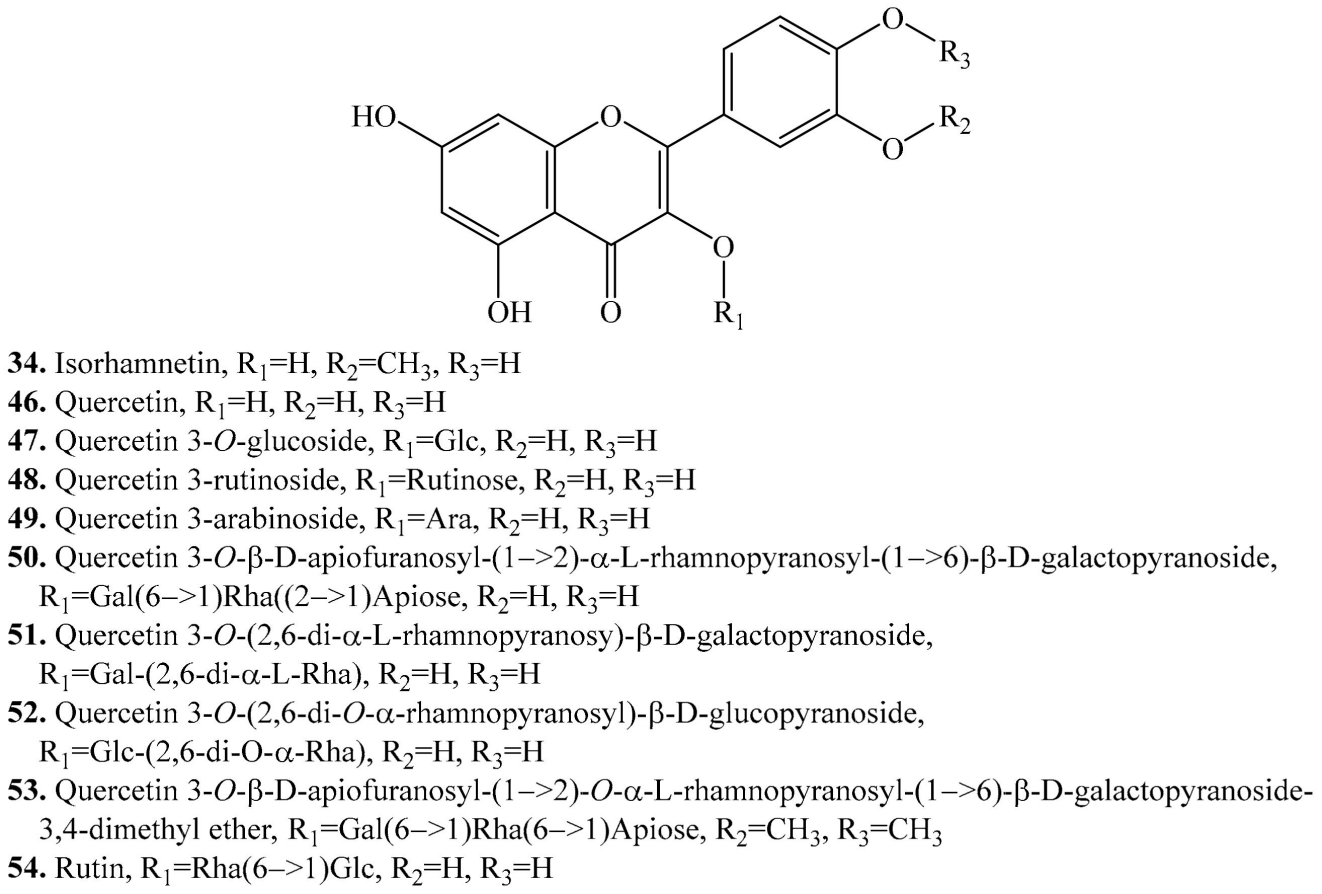
Structures of the flavonols isolated from quinoa.

About 21 flavonols have been identified in quinoa. Most of them are present in the seeds. Their biological activities are listed in Table 4, and their structures are shown in Figure 4.

Both kaempferol (**35**) and quercetin (**46**) are two main flavonols. They are in the form of glycosides present in quinoa. Structure-activity relationship of their antioxidant activity showed that the ability to quench free hydroxyl radicals increased with the amount of hydroxyl groups in the ring B. For example, myricetin (**45**) was a stronger antioxidant than kaempferol (**35**) [118]. In addition, the compounds with 3’,4’-dihydroxy substituents in the ring B had much stronger antioxidative activities than those without *ortho*-dihydroxy substitution in the ring B [119]. Quercetin (**46**) was the strongest antioxidant among the flavonoids. Both isorhamnetin (**34**) and kaempferol (**35**) were the most abundant flavonoids in quinoa leaves, and it also contained large amounts of rutin (**54**) [27]. Four kaempferol 3-glycosides (**38**–**41**) exhibited moderate antioxidant activity while two quercetin 3-glycosides (**50**,**51**) showed strong antioxidant activity, suggesting that quinoa could represent an important source of free radical inhibitors [120].

Many flavonoids are characterized by antibacterial, antifungal and antiviral activities, not only against plant pathogens, but also against the pathogens for humans and animals (Table 4). Kaempferol (**35**) and its derivatives showed antibacterial activity against Gram-positive and Gram-negative bacteria, as well as against the fungus *Candida glabrata* [121,122].

About eight quercetin derivatives (**46**–**53**) have been identified in quinoa. Kaempferol (**35**), myricetin (**45**) and quercetin (**46**) acted as the deterrents against *Radopholus similis* and *Meloidogyne incognita* [82]. Quercetin-3-glucoside (**47**) and rutin (**54**) from *Pinus banksiana* inhibited the development of *Lymantria dispar* and increased its mortality [123]. Quercetin (**46**), quercetin 3-*O*-glucoside (**47**) and its six derivatives exhibited inhibitory activity on the shoot growth of *Arabidopsis thaliana* as well as on the spore germination of the fungus *Neurospora crassa* [124].

### 3.3. Flavanones and Their Biological Activities or Functions

**Figure 5:**
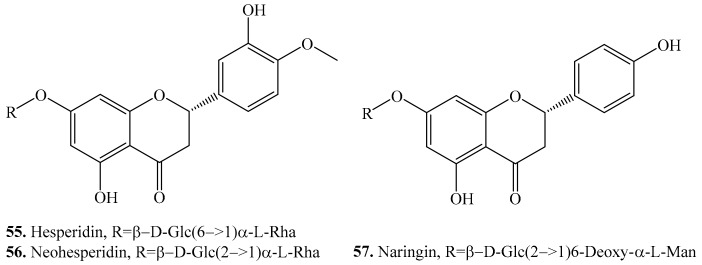
Structures of the flavanones isolated from quinoa.

Three flavanones hesperidin (**55**), neohesperidin (**56**), and naringin (**57**) were identified in quinoa seeds (Table 5 and Figure 5). Both hesperidin (**55**) and neohesperidin (**56**) were found in the sprouts [34]. These flavanones isolated from other plant species were screened to show a variety of biological activities such as neuroprotective [147], antioxidant [157], anti-inflammatory [158] and antifungal [159] activities.

### 3.4. Flavanols and Their Biological Activities or Functions

**Figure 6:**
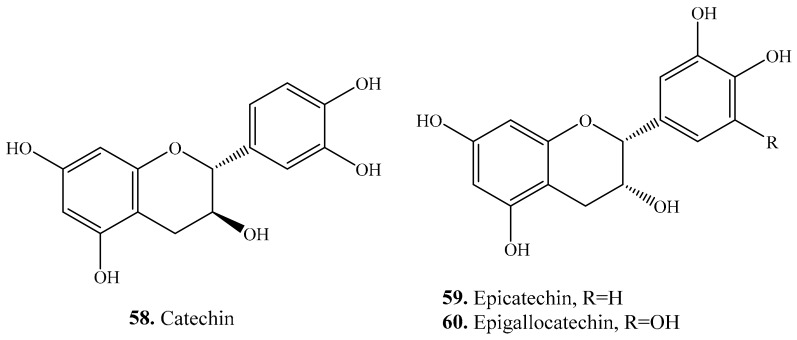
Structures of the flavanols isolated from quinoa.

Three flavanols namely catechin (**58**), epicatechin (**59**), and epigallocatechin (**60**) were found in quinoa seeds. Their biological activities are listed in Table 6, and their structures are shown in Figure 6. They generally showed antioxidant [149,168] and antimutagenic [169] activities.

### 3.5. Isoflavones and Their Biological Activities or Functions

**Figure 7:**
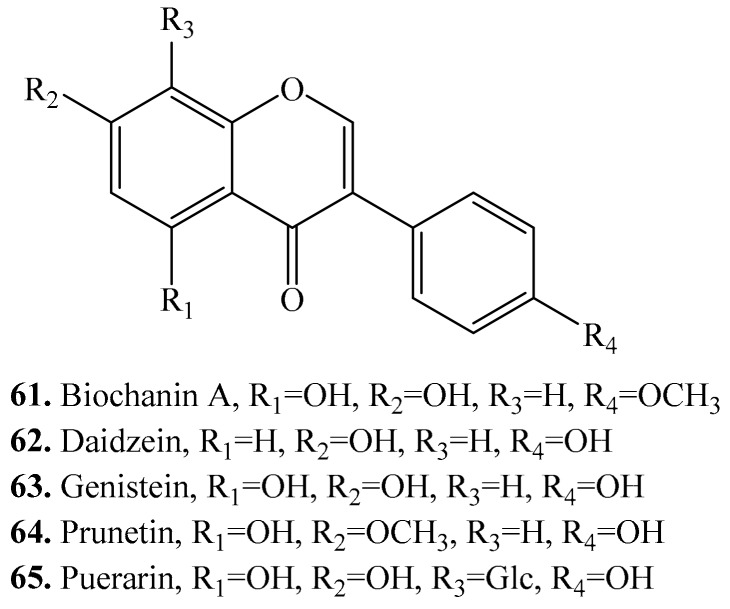
Structures of the the isoflavonoids isolated from quinoa.

Five isoflavanones, i.e., biochanin (**61**), daidzein (**62**), genistein (**63**), prunetin (**64**), and puerarin (**65**) were found in quinoa (Table 7 and Figure 7). They showed antinematodal activities on *Radopholus similis* [82]. Isoflavones are recognized to be estrogenic compounds that are often associated with a reduced risk of cancers. The estrogenic activity can be enhanced after metabolization to more active compounds such as daidzein (**62**) and genistein (**63**) by gut microorganisms [175].

## 4. Terpenoids and Their Biological Activities or Functions

The terpenoids in quinoa mainly include monoterpenoids and triterpenoids which are biosynthesized through the isoprenoid metabolic pathway. The monoterpenoids usually play functions as allelochemicals in quinoa. The triterpenoids are present in the seed coats (also called bran or hull), and have a characteristic bitter or astringent taste to protect it from birds and insects, and possess detergent properties [2]. The saponins are also of interest as valuable adjuvants and the first saponin-based vaccines have been introduced commercially [203].

### 4.1. Monoterpenoids and Their Biological Activities or Functions

**Figure 8:**
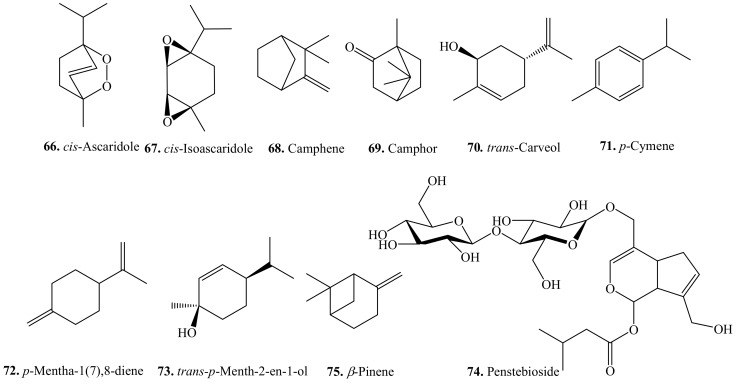

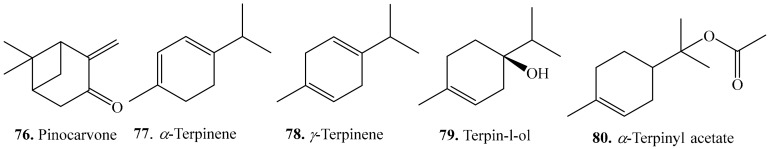
Structures of the monoterpenoids isolated from quinoa.

Quinoa monoterpenoids and their biological activities are listed in Table 8. Their structures are shown in Figure 8. At least 15 monoterpenoids in the essential oils of quinoa from the East Mediterranean have been identified [204]. Penstebioside (**74**) was an iridoid glycoside isolated from the flour of quinoa [33]. γ-Terpinene (**78**) was also isolated from rice to show antibacterial activity on *Xanthomonas oryzae* pv. *oryzae* (*Xoo*) [205].

### 4.2. Sesquiterpenoids and Their Biological Activities or Functions

**Figure 9:**
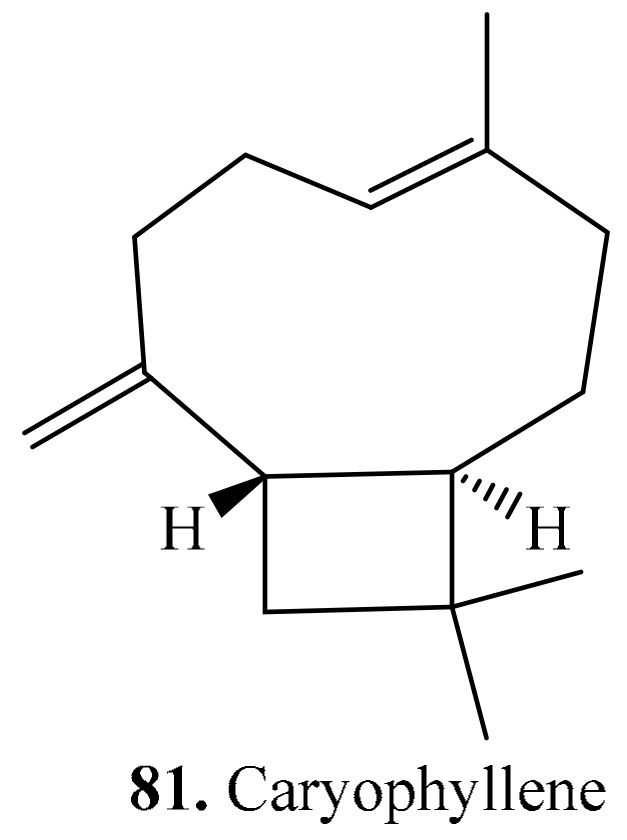
Structure of the sesquiterpenoid isolated from quinoa.

Only one sesquiterpene namely caryophyllene (**81**) was identified in quinoa [204]. Its structure is shown in Figure 9.

### 4.3. Triterpenoids and Their Biological Activities or Functions

Triterpenoids, including their aglycones (sapogenins) and glycosides (saponins), are mainly present in the bran to protect quinoa from pests and herbivores (i.e., birds and insects) and pathogenic microorganisms [206]. Quinoa saponins are characterized as the bitter metabolites. The quinoa could be classified into bitter and sweet varieties according to the triterpenoid saponin content, which is much lower in the sweet varieties and higher in the bitter ones [138,207].

The crude saponin fraction inhibited the growth of *Candida albicans* at 50 μg/mL [208]. The alkali-transformed saponin from quinoa bran showed inhibition against halitosis-related bacterium *Fusobacterium nucleatum*, with a minimum inhibitory concentration (MIC) of 31.3 μg/mL. It could be used as an antibacterial agent to treat halitosis [209]. When the fungal pathogen *Botrytis cinerea* was treated with the saponin extracts, mycelial growth and conidial germination were significantly inhibited [210].

When golden apple snails (*Pomacea canaliculata*, GAS) were treated with the crude saponin under laboratory conditions in 24 h at approximately 33 μg/mL, they were completely killed [211]. Similarly, when giant apple snails (*Pomacea maculata*) were treated with saponins above 7 μg/mL after 72 h, they were also 100% killed. Quinoa saponin could be a viable product to safely control *P. maculata* in rice fields [212]. Therefore, quinoa saponins could be developed into molluscicide. In addition, this molluscicide was found to be non-toxic to other non-target species such as goldfish (*Carassius auratus*) and tilapia (*Oreochromis mossambicus*), while providing adequate protection from *Pomacea* snails to newly sprouted rice seeds under laboratory conditions [211,213].

**Figure 10:**
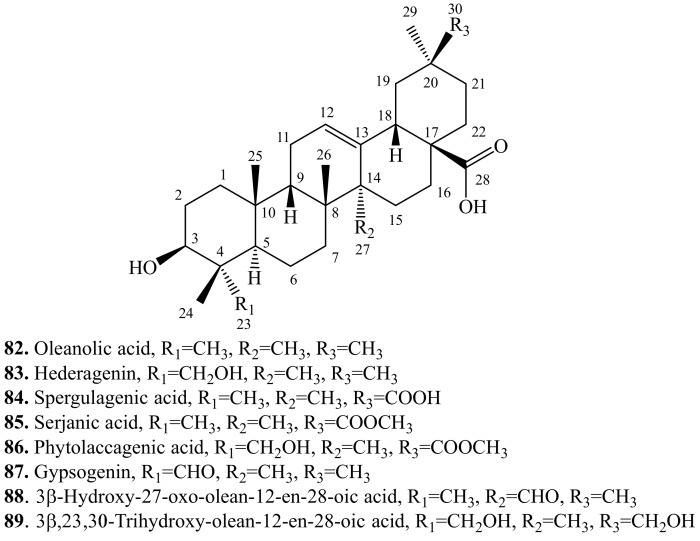
Structures of the main triterpenoid aglycones in quinoa.

The quinoa triterpenoids contain either tetracycles or pentacycles in their core structures. Most of them are pentacyclic triterpenoids in the form of saponins. The saponins contain an aglycone (sapogenin) and one to three saccharide chains in their structures, and were classified according to the number of saccharide chains as mono-, di-, and tridesmosides. Liquid chromatography-tandem mass spectrometry (LC-MS/MS) allowed a complete preassignment and identification of the major saponins and aglycones [214]. The main aglycones (Figure 10), which are oleanolic acid (**82**), hederagenin (**83**), spergulagenic acid (**84**), serjanic acid (**85**), phytolaccagenic acid (**86**), gypsogenin (or named 3β-hydroxy-23-oxo-olean-12-en-28-oic acid) (**87**), 3β-hydroxy-27-oxo-olean-12-en-28-oic acid (**88**), and 3β,23,30-trihydroxy-olean-12-en-28-oic acid (**89**), and their glycosides are shown in Table 2, Table 3, Table 4, Table 5, Table 6, Table 7, Table 8 and Table 9 [23,215,216,217]. They have a five-ring skeleton, and are biosynthesized from *β*-amyrin *in planta* (**134**) [23]. Among them, oleanolic acid is the major aglycone [218]. Sugars, which were glucose (Glc), glucuronic acid (GlcA), galactose (Gal), arabinose (Ara), and xylose (Xyl), can be linked to the aglycone at C-3, C-23 or C-28 [214].

#### 4.3.1. Oleanolic Acid Derivatives and Their Biological Activities or Functions

**Figure 11:**
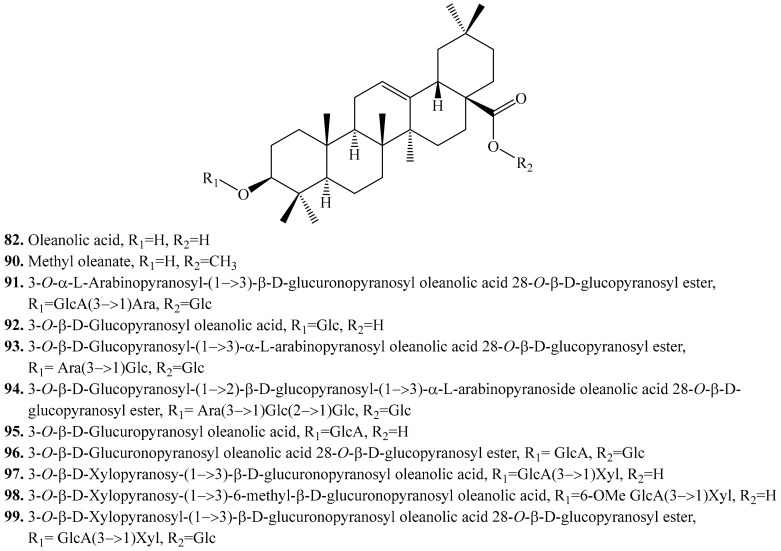
Structures of the oleanolic acid and its glycosides isolated from quinoa.

About 11 oleanolic acid analogues have been identified in quinoa. Their biological activities are listed in Table 9, and the structures are shown in Figure 11. The major sugars of the saccharide moieties are arabinose, glucose and galactose [219].

Oleanolic acid and its glycosides are mainly present in the bran (seeds) of quinoa. They showed a variety of biological activities such as antimicrobial [220,221], anti-HIV [222], anti-inflammatory [223,224], antioxidant [225], antifertility [226], antitumor or anticancer [227,228,229], antidiabetogenic [230], anticomplement [231] properties. They also exhibited inhibitory activities on serin protease and porcine pancreatic elastase [232].

#### 4.3.2. Hederagenin Derivatives and Their Biological Activities or functions

**Figure 12:**
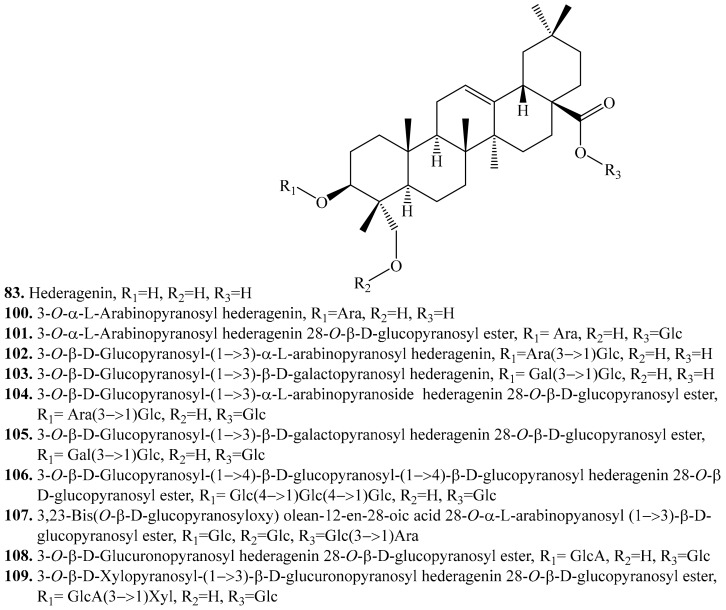
Structures of hederagenin and its glycosides isolated from quinoa.

About 10 hederagenin analogues have been identified in quinoa. Their biological activities are listed in Table 10, and the structures are shown in Figure 12. Hederagenin (**83**) was the main aglycone of saponins from quinoa leaves [239]. Hederagenin glycosides existed in nature and possessed many biological activities such as molluscicidal [240], cytotoxic [241], antifungal [242], leishmanicidic [243], anti-inflammatory [244] activities, and they have been recently reported to show low cytotoxic properties for several human cancer cell lines with median effective concentration (EC_50_) >30 μM [245]. Hederagenin monodesmosides also showed strong haemolytic activity [208], hence the saponins have been considered as the serious antinutritional factors [246]. Hederagenin from the leaves of ivy (*Hedera helix*) induced apoptosis of LoVo cells through the mitochondrial apoptotic pathway, which indicated that hederagenin might be a promising therapeutic candidate for the prevention and treatment of human colon cancer [247].

#### 4.3.3. Spergulagenic Acid Derivatives and Their Biological Activities or Functions

**Figure 13:**
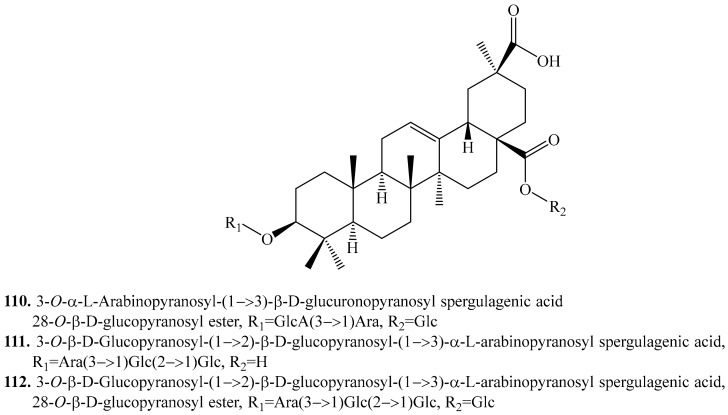
Structures of the spergulagenic acid glycosides isolated from quinoa.

Spergulagenic acid (**84**), a pentacyclic triterpene used in medicine, was found in diverse plant families [258]. Until now, three spergulagenic acid glycosides (Table 11) were identified in quinoa [217,256], though spergulagenic acid as the aglycone has not been isolated from quinoa. Their structures are shown in Figure 13.

#### 4.3.4. Serjanic Acid Derivatives and Their Biological Activities or Functions

**Figure 14:**
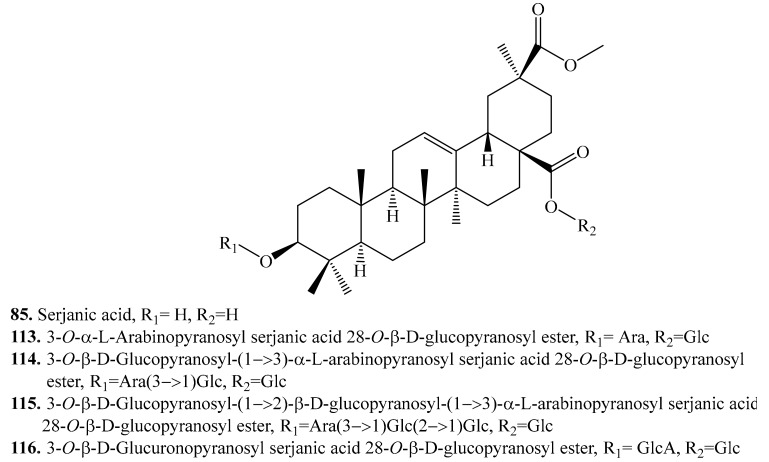
Structures of serjanic acid and its glycosides isolated from quinoa.

Serjanic acid (**85**) is the aglycone with only the bidesmosides to be found in quinoa [217]. About 5 serjanic acid analogues have been identified in quinoa (Table 12, Figure 14). Hemolysis tests showed that most monodesmoside saponins were active, and most bidesmoside saponins were inactive as the monodesmosides can reduce hydrophobic interactions with membrane lipids [208]. Similarly, both 3-*O*-α-l-arabinopyranosyl serjanic acid 28-*O*-β-d-glucopyranosyl ester (**113**) and 3-*O*-β-d-glucuronopyranosyl serjanic acid 28-*O*-β-d-glucopyranosyl ester (**116**) had weaker hemolytic activity (IC_50_ > 100 μg/mL) than their sapogenin (serjanic acid, IC_50_ = 50 μg/mL) [11].

#### 4.3.5. Phytolaccagenic Acid Derivatives and Their Biological Activities or Functions

**Figure 15:**
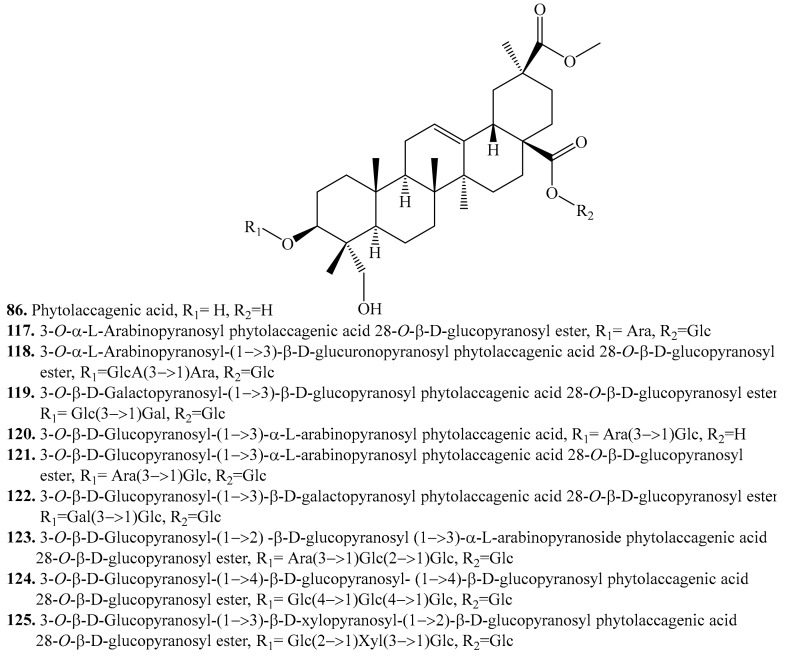
Structures of phytolacagenic acid and its glycosides isolated from quinoa.

Phytolaccagenic acid (**86**) might be originated from serjanic acid (**85**) by subsequent oxidative enzymatic steps involving the formation of the corresponding alcohol substituted at C-23 *in planta* [11]. It is one of the main structures of quinoa sapogenins. About 10 phytolaccagenic acid analoques have been identified in quinoa. They are listed in Table 13, and the structures are shown in Figure 15.

Phytolaccagenic acid saponins are highly concentrated in the bran (seed coats), which are more exposed to water during germination compared to oleanolic acid saponins [259]. It was suggested that a short saccharide chain (1 or 2 glycosyl residues) requires the presence of an additional longer one to make the saponin water-soluble [260]. Phytolaccagenic acid was employed as the anti-inflammatory drug of oral administration [234].

#### 4.3.6. Gypsogenin Derivatives and Their Biological Activities or Functions

**Figure 16:**
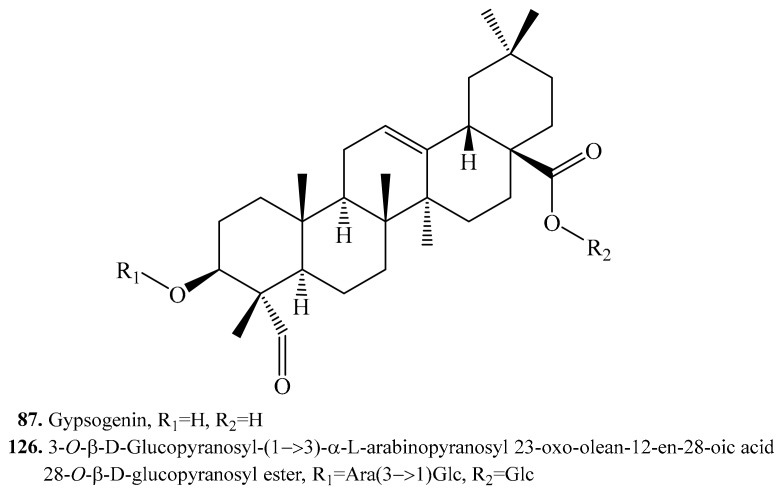
Structures of the gypsogenin derivatives isolated from quinoa.

Gypsogenin (or named 3β-hydroxy-23-oxo-olean-12-en-28-oic acid) (**87**) and its glycoside 3-*O*-β-d-glucopyranosyl-(3)-α-l-arabinopyranosyl 23-oxo-olean-12-en-28-oic acid 28-*O*-β-d-gluco-pyranosyl ester (**126**) were isolated from quinoa (Table 14, Figure 16). They showed cytotoxic activity [11].

#### 4.3.7. 3β-Hydroxy-27-oxo-olean-12-en-28-oic Acid Derivatives and Their Biological Activities or Functions

**Figure 17:**
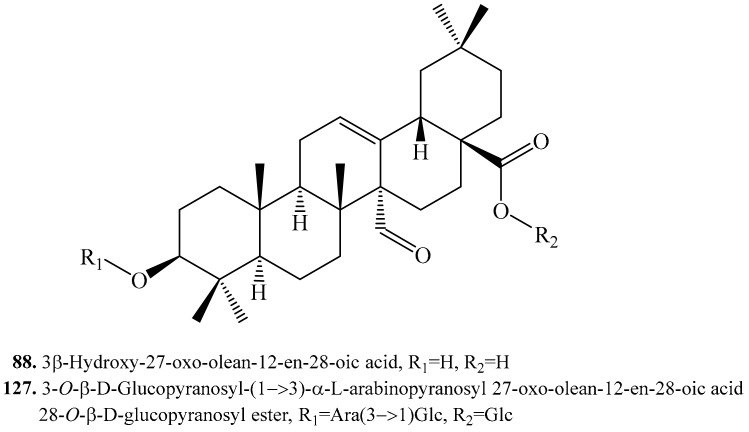
Structures of the 3β-hydroxy-27-oxo-olean-12-en-28-oic acid triterpenoids isolated from quinoa.

3β-Hydroxy-27-oxo-olean-12-en-28-oic acid (**88**) and its glycoside 3-*O*-β-d-glucopyranosyl-(1→3)-α-l-arabinopyranosyl 27-oxo-olean-12-en-28-oic acid 28-*O*-β-d-glucopyranosyl ester (**127**) were isolated from quinoa (Table 15, Figure 17). 3β-Hydroxy-27-oxo-olean-12-en-28-oic acid (**88**) showed same cytotoxic effect as 3β-hydroxy-23-oxo-olean-12-en-28-oic acid (**87**) with an IC_50_ value of 25.4 μg/mL. This suggests that the CHO groups at C-23 or C-27 are correlated with the increased cytotoxicity [11].

#### 4.3.8. 3β,23,30-Trihydroxy-olean-12-en-28-oic acid Triterpenoids and Their Biological Activities or Functions

**Figure 18:**
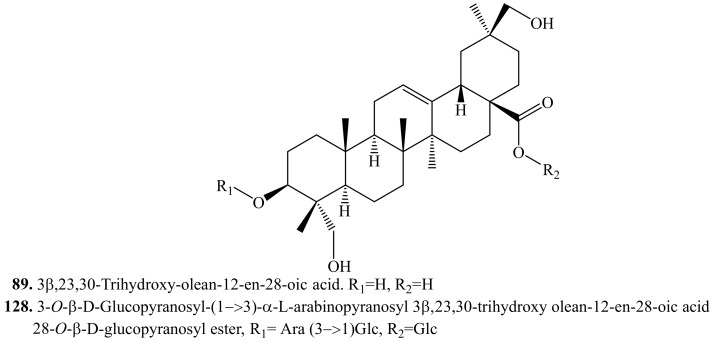
Structures of the 3β,23,30-trihydroxy-olean-12-en-28-oic acid triterpenoids isolated from quinoa.

3β,23,30-Trihydroxy-olean-12-en-28-oic acid (**89**) and 3-*O*-β-d-glucopyranosyl-(1→3)-α-l-arabinopyranosyl 3β,23,30-trihydroxy olean-12-en-28-oic acid 28-*O*-β-d-glucopyranosyl ester (**128**) have been isolated from quinoa (Table 16, Figure 18). Hederagenin (**83**) was considered as the substrate for the production of 3β,23,30-trihydroxyolean-12-en-28-oic acid (**89**), following a mechanism involving a stereochemically specific enzyme able to insert one hydroxyl group into the C-30 position of the triterpene skeleton [11].

#### 4.3.9. Other Triterpenoids and Their Biological Activities or Functions

**Figure 19:**
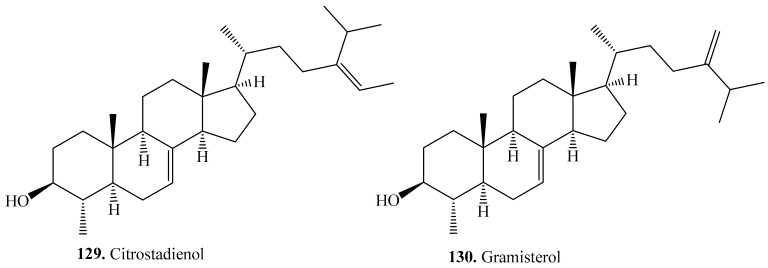

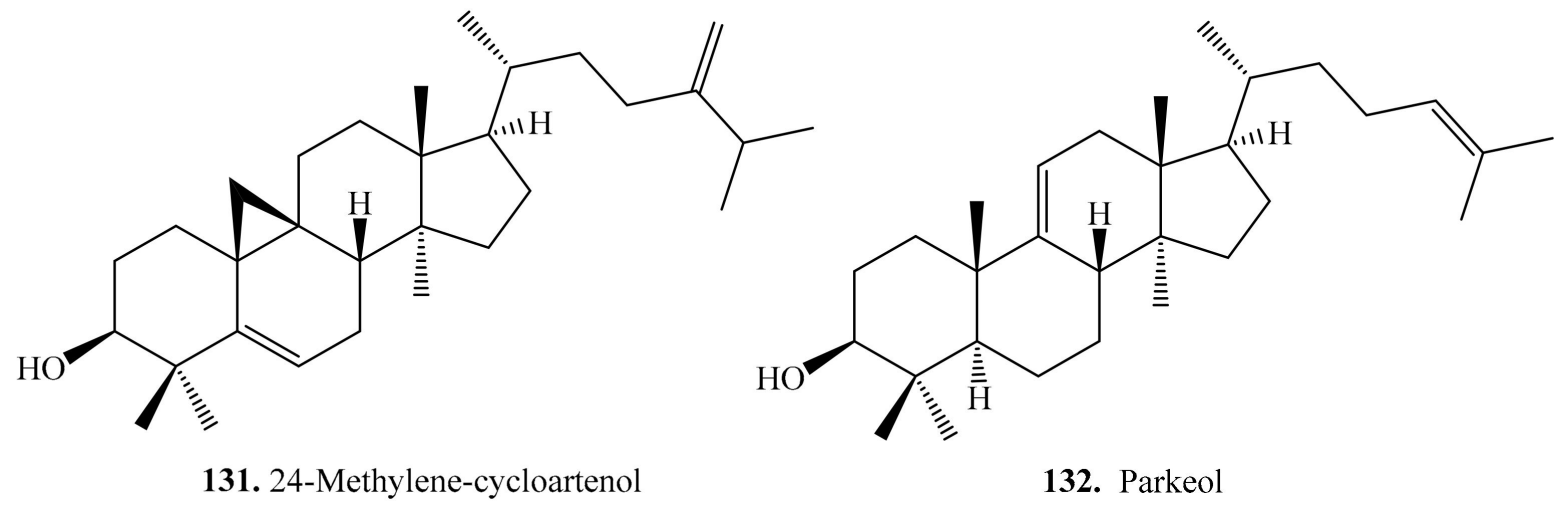
Structures of the tetracyclic triterpenoids isolated from quinoa.

**Figure 20:**
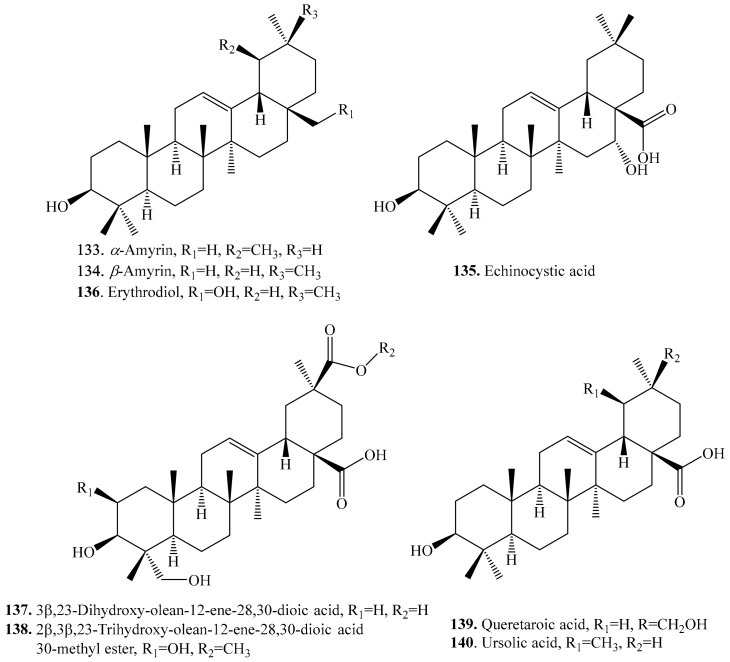
Structures of the other pentacyclic triterpenoids isolated from quinoa.

Other triterpenoids include tetracyclic and pentacyclic triterpenoids. Their biological activities are shown in Table 17, and the structures are shown in Figure 19 and Figure 20.

Four tetracyclic triterpenoids including two nortriterpenoids citrostadienol (**129**) and gramisterol (**130**) have been isolated from quinoa seeds [261]. Citrostadienol (**129**) showed anticomplementary activity [262], and gramisterol (**130**) showed anti-cancer activity [8].

Among the other pentacyclic triterpenoids, β-amyrin (**133**) was considered as the precursors of other triterpenoids in their biosynthetic pathways [23].

### 4.4. Meroterpenoids and Their Biological Activities or Functions

**Figure 21:**
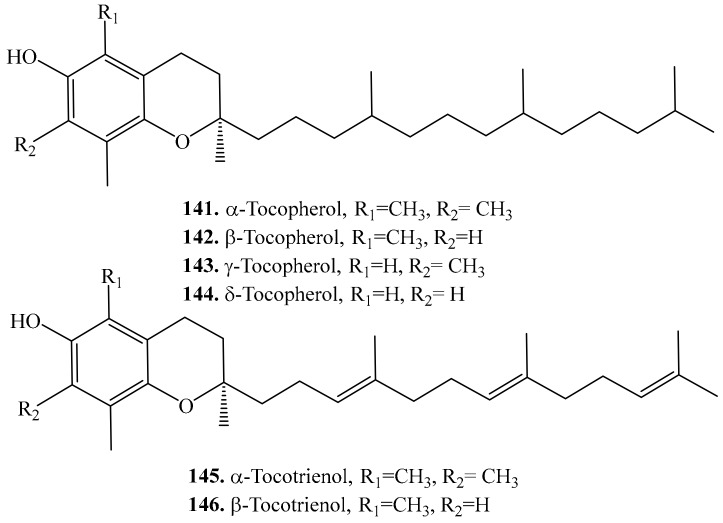
Structures of the meroterpenoids isolated from quinoa.

Mertoterpenoids are natural products of mixed biosynthetic origin which are partially derived from terpenoids. Meroterpenoids were also found in quinoa that include tocopherols (**141**–**144**) and tocotrienols (**145**,**146**). Their biological activities are listed in Table 18, and the structures are shown in Figure 21.

The total tocopherol content ranged from 37.49 to 59.82 μg/g [275]. All four tocopherol isoforms (α, β, γ, and δ) have been detected in quinoa seeds, with γ-tocopherol (**143**) to be the most abundant followed by α-tocopherol (**141**), β-tocopherol (**142**) and δ-tocopherol (**144**) was the least [276]. Tocopherols acted as strong antioxidants and had many essential physiological functions such as anticoagulant, essential regulator of metabolic processes including inflammation and cancer in humans [277,278]. Among 4 tocopherols homologues, α-tocopherol (**141**) was considered a stronger antioxidant, whereas γ-tocopherol (**143**) was a stronger anti-inflammatory agent [279,280]. γ-Tocopherol (**143**) was the main lipophilic tocopherol in quinoa [281].

Both α-tocotrienol (**145**) and β-tocotrienol (**146**) were also identified in quinoa seeds [275]. They were the members of the vitamin E family to show antioxidant and anti-inflammatory properties [282].

## 5. Steroids and Their Biological Activities or Functions

Quinoa contains a lot of biologically active phytoecdysteroids, which have been implicated in plant defense from insects, and have displayed potential pharmacologic and metabolic properties in mammals. According to the carbon skeletons, quinoa steroids can be classified as C_27_-, C_28_- and C_29_-steroids.

About 36 steroids have been identified in quinoa. Seven sterols were identified among the quinoa lipids, namely cholesterol (**147**), campesterol (**160**), Δ^7^-campesterol (**161**), Δ^5^-avenasterol (**172**), β-sitosterol (**176**), stigmasterol (**181**), and Δ^7^-stigmasterol (**182**) [285]. Eleven 4,4-desmethylsterols were assigned, with Δ^7^-avenasterol (**173**), β-sitosterol (**176**), and Δ^7^-stigmastenol (**180**) being the most abundant (8.7, 27.2, and 51.3% of total sterols, respectively) [261].

### 5.1. C_27_-Steroids and Their Biological Activities or Functions

**Figure 22:**
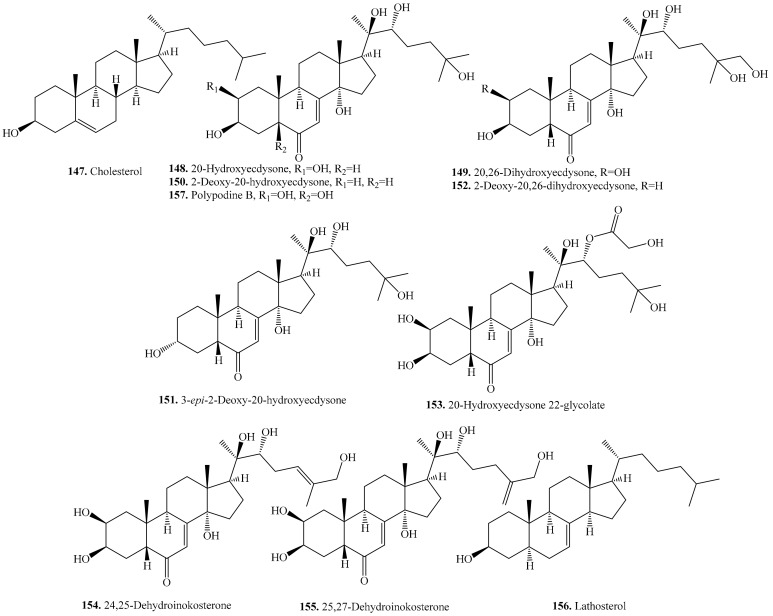
Structures of the C_27_-steroids isolated from quinoa.

Eleven C_27_-steroids were identified in quinoa seeds which are listed in Table 19. Their structures are shown in Figure 22. Among them, ecdysteroids are main steroids which are insect moulting hormones and protect plants against non-adapted insects and nematodes [21]. Ecdysteroids are mainly present in the bran, the major component is 20-hydroxyecdysone (**148**) possessing a 14α-hydroxy-7-en-6-one chromophore and A/B-*cis* ring fusion (5β-H) [21].

Eating quinoa seeds or quinoa-derived products provides significant amounts of ecdysteroids that may be beneficial to animal or human health [22]. Quinoa extract enriched in 20-hydroxyecdysone has an antiobesity activity in vivo and could be used as a nutritional supplement for the prevention and treatment of obesity and obesity-associated disorders. The findings indicated that the extract acted by reducing both fatty acid uptake and esterification in adipocyte [286]. It was found that 8 isolated ecdysteroids showed a stronger free-radical-scavenging activity, which was almost 3 to 8 times higher than that of the well-known antioxidant compound, BHA, and also possessed a strong ability to sequester ferrous ions. This observation supported that if the number of hydroxyl and methyl groups bearing the carbon skeleton of ecdysteroids is higher, the antioxidant activity becomes stronger. The ability of ecdysteroids to sequester ferrous ions is thought to be due to their carbonyl conjugated to a double bond attached to the C-7. Ecdysteroids are also able to inhibit skin collagenase, and could therefore also prevent skin ageing [287]. In addition, ecdysteroids have been reported to occur in Chenopodiaceae to show their possible chemotaxonomic and ecological implications [21].

### 5.2. C_28_-Steroids and Their Biological Activities or Functions

**Figure 23:**
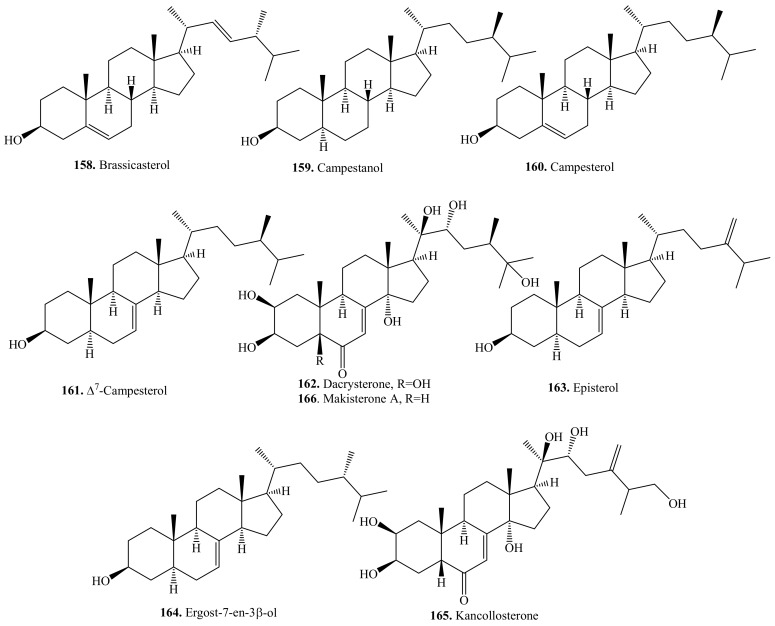

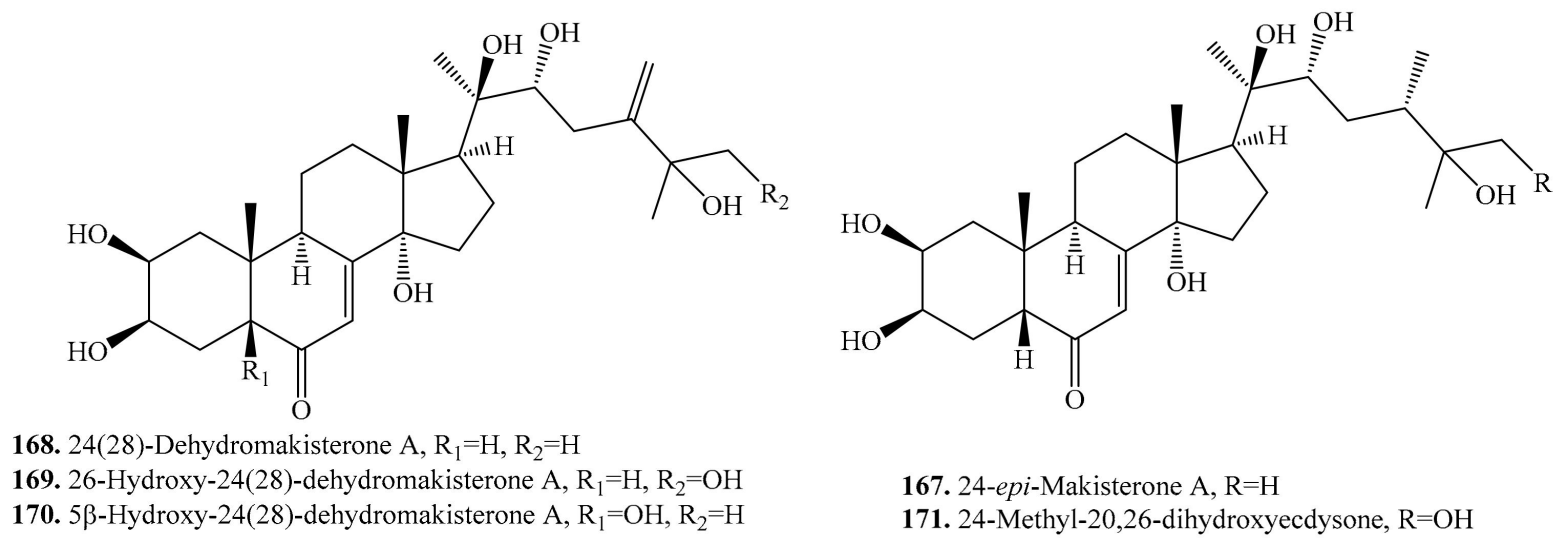
Structures of the C_28_-steroids isolated from quinoa.

About 14 C_28_-steroids such as campesterol (**160**), makisterone A (**166**), and their derivatives have been identified from quinoa seeds [34]. Their biological activities are listed in Table 20, and the structures are shown in Figure 23. The main biological activities include antioxidant activity [289], antiangiogenic activity [291], and inhibitory activity on collagenase [287].

### 5.3. C_29_-Steroids and Their Biological Activities or Functions

**Figure 24:**
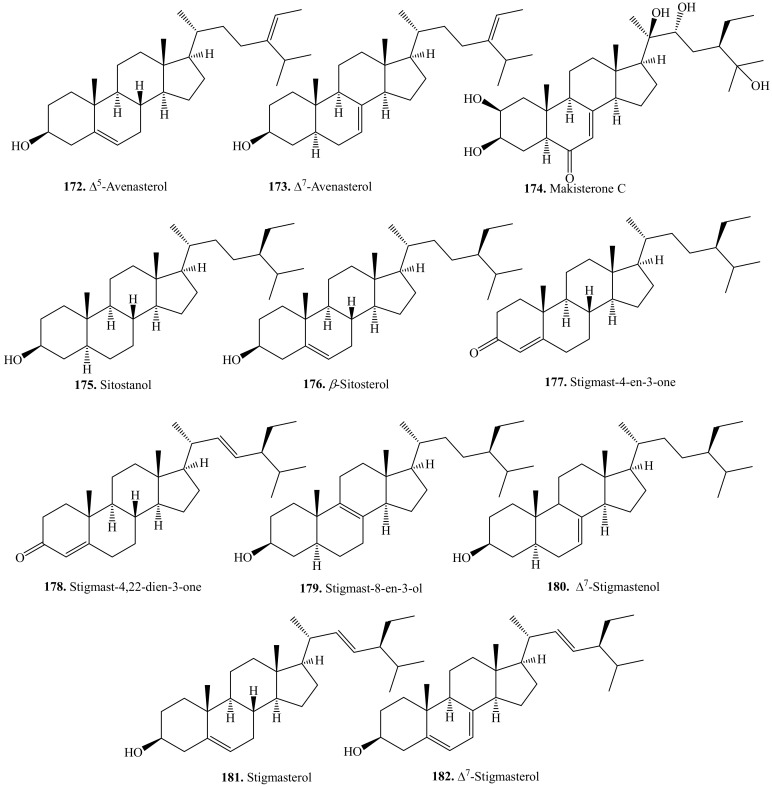
Structures of the C_29_-steroids isolated from quinoa.

The main C_29_-steroids in quinoa included avenasterol (**172**/**173**), sitosterol (**176**), stigmasterol (**181**), and their derivatives. They were all identified in the lipid extract of quinoa seeds [261]. Their biological activities are listed in Table 21, and the structures are shown in Figure 24.

β-Sitosterol (**176**) has been reported to have a variety of biological activities such as anti-inflammatory [294], antioxidant [295], and antidiabetic [296] activities. Stigmasterol (**181**) also exhibited various biological activities such as anti-inflammatory [297], anti-tumor [298], antifungal [299], anti-hypercholestrolemic [300], and cytotoxicity [301] activities.

## 6. Nitrogen-Containing Metabolites and Their Biological Activities or Functions

**Figure 25:**
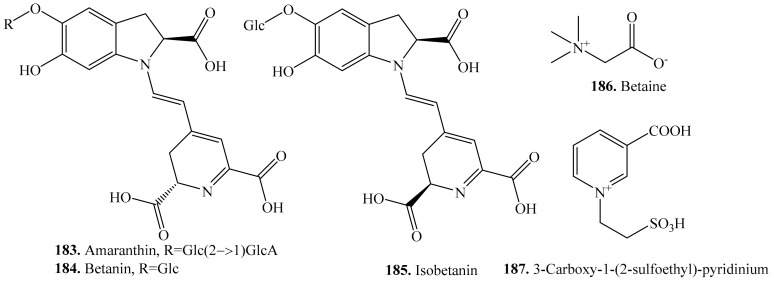

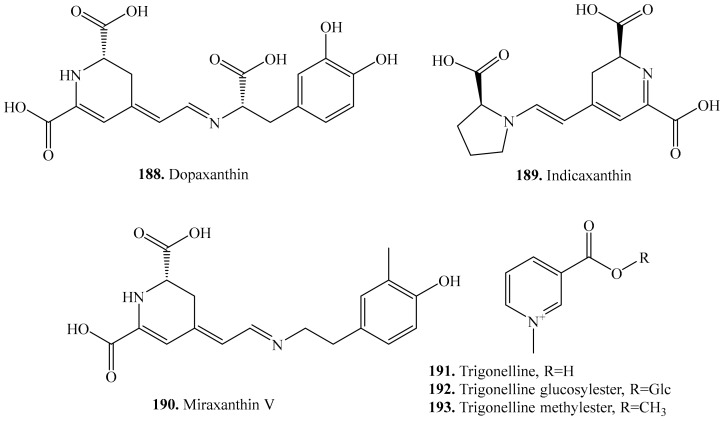
Structures of the nitrogen-containing metabolites isolated from quinoa.

About 12 nitrogen-containing metabolites have been identified in quinoa seeds. They belong to the derivatives of glycine and tyrosine. Their biological activities are listed in Table 22, and the structures are shown in Figure 25.

Betalains are tyrosine-derived red-violet and yellow pigments found in quinoa [311]. They are divided into two groups, betacyanins (red and purple) and betaxanthins (yellow and orange). Betacyanins are derivatives of betanidin, the conjugate of betalamic acid with *cyclo*-Dopa. Betacyanins, including amaranthin (**183**), betanin (**184**) and isobetanin (**185**), were confirmed in red and black quinoa seeds, instead of anthocyanins [35]. Betaxanthins are conjugates of betalamic acid with amino acids. Betaxanthins mainly include dopaxanthin (**188**), indicaxanthin (**189**), and miraxanthin V (**190**) in quinoa [312].

Betalains showed promising bioactive potential, such as high antioxidant and free radical scavenging activities [30]. Betacyanins and betaxanthins showed the highest antioxidant activity by comparing the white and black quinoa varieties [35]. These two varieties are characterized by a high content of dopaxanthin (**188**), whose dihydroxylated substructure demonstrated high antioxidant capacity [36].

Other nitrogen-containing metabolites in quinoa include betaine (**186**), trigonelline (**191**), and their derivatives. In mammals, betaine (**186**) acted as an osmolyte in the inner medulla of the kidney, preserving osmotic equilibrium, maintaining at the same time the tertiary structure of macromolecules [313]. Trigonelline (**191**) was considered to be an important multifunctional natural plant hormone with potential taxonomic value [314], and has been shown to stabilize enzyme activity in vitro [315].

## 7. Conclusions and Future Perspectives

This review focuses on the structures, isolation parts, biological activities or functions of quinoa secondary metabolites during the past 40 years. Flavonoids and phenolic acids were mostly derived from quinoa seeds. Steroids were mostly separated from quinoa bran. Triterpenoids were also mainly located in the bran. Their biological activities or functions have been reported but not comprehensive, and are needed to be systematically evaluated in the future.

The bitter taste associated with saponins (triterpenoids) greatly limits the use of quinoa as food [18]. Approximately 34% of quinoa saponins are present in the bran, indicating that dehulling could remove almost one half of the saponins. The seeds should be milled to remove the bran (seed coats) to make them edible [239]. Another method to remove saponins from the seeds is washing due to the high water solubility of saponins although this method can lead to the loss of some nutrients such as vitamins and minerals [18].

With the increased demand for quinoa, the problem that comes with it is that the bran is discarded as an industrial production waste. In order to increase the added value of quinoa, the bran (seed coat) should be fully exploited and utilized [321]. Quinoa saponins have shown their great potential applications. They can be used in the pharmaceutical industry as the saponins can induce changes in intestinal permeability which can be useful for the absorption of specific medicines and in hypocholesterolemia [15,322,323,324]. Quinoa saponins are also of interest as valuable adjuvants and the first saponin-based vaccines have been introduced commercially [203]. In addition, the saponins can be used as bitters, antibiotics to control pathogenic fungi and bacteria, or to protect crop against attack by birds and other pests [325]. Quinoa saponins have been successfully developed as a bioinsecticide in Bolivia [326]. They can also be used as emulsifiers and detergents due to surface active characteristics which saponins have [327]. Quinoa saponins might be developed into products like soaps, shampoos, and bitters in the future. As phenolic acids, flavonoids, and steroids are also abundant in the bran, they can be developed into antimicrobials, antioxidants, and insect moulting hormones, respectively [5,21]. It is worth mentioning that 20-hydroxyecdysone (**148**), mainly present in the bran, has potential for development as an insect moulting hormone [21]. After the above secondary metabolites are extracted from the bran, the remaining residues, which mainly contain cellulose, could be either used as feed, or femented into biofuels and biofertilizer.

Biosynthesis research on quinoa secondary metabolites has rarely been reported. Methyl jasmonate was reported to induce accumulation of saponins in quinoa leaves and induce the expression of saponin biosynthetic genes in quinoa [328]. Knowledge of the saponin biosynthesis and its regulation in quinoa may aid the further development of sweet cultivars. Genome sequencing of quinoa revealed a diversity of biosynthetic core genes of secondary metabolites [329], indicating the great potential of this plant to produce various secondary metabolites with biological activities or functions which merit further investigation.

## Figures and Tables

**Table 1:** Benzoic acid analogues and their biological activities or functions.

Name	Quinoa Part Used for Isolation	Biological Activity or Function	Ref.
Benzoic acid (**1**)	Leaves and flour	-	[27,33]
4-Hydroxybenzoic acid = *p*-Hydroxybenzoic acid (**2**)	Seeds	-	[25]
	Leaves and seeds	-	[27,34]
		Antimicrobial activity	[28]
		Allelopathic effect	[30]
2,4-Dihydroxybenzoic acid (**3**)	Seeds	-	[25,35]
2,5-Dihydroxybenzoic acid (**4**)	Seeds	-	[35]
3,4-Dihydroxybenzoic acid (**5**)	Seeds	-	[35]
Canthoside A (**6**)	Flour	-	[33]
Ethyl-*m*-digallate (**7**)	Flour	-	[33]
		Antifeedant activity	[32]
Gallic acid (**8**)	Leaves, sprouts and seeds	-	[27,34]
		Antioxidant activity	[31]
		Antibacterial activity	[36]
1-*O*-Galloyl-β-d-glucoside (**9**)	Seeds and flour	-	[33]
Protocatechuic acid (**10**)	Sprouts and seeds	-	[25,37]
		Antioxidant activity	[31]
		Anticancer activity	[38]
		Antibacterial activity	[39]
		Antiulcer activity	[40]
		Antiageing activity	[41]
		Anti-inflammatory, antiibrotic, antiatherosclerotic, hyperlipidemic, analgesic, hepatoprotective and nephroprotective activities	[42]
		Antiviral activity	[43]
Protocatechuic acid 4-*O*-glucoside (**11**)	Flour	-	[33]
		Antioxidant activity	[44]
Syringic acid (**12**)	Leaves and seeds	-	[25,26]
		Allelopathic effect	[30]
		Antioxidant activity	[31]
		Antimicrobial activity	[45]
		Hepatoprotective effect	[46]
		Anti-inflammatory activity	[47]
Vanillic acid (**13**)	Leaves and seeds	-	[25,34]
		Allelopathic effect	[30]
		Hepatoprotective effect	[46]
		Antioxidant and antimicrobial activities, and inhibitory activity on COX-I and COX-II	[48]
Vanillic acid glucosyl ester (**14**)	Seeds	-	[49]
Vanillic acid 4-*O*-glucoside (**15**)	Seeds	-	[35]
Vanillin (**16**)	Seeds and flour	-	[25,33,35]
		Antioxidant activity	[50]
		Antimicrobial activity	[51]
		Antidepressant activity	[52]
		Anti-angiogenic, anti-inflammatory and anti-nociceptive activities	[53]

**Table 2:** Cinnamic acid analogues and their biological activities or functions.

Name	Quinoa Part Used for Isolation	Biological Activity or Function	Ref.
Caffeic acid (**17**)	Seeds	-	[25,34]
		Antimicrobial activity	[29]
		Allelopathic effect	[30]
		Antioxidant activity	[31]
		Anti-apoptotic activity	[55]
		Inhibitory activity on xanthine oxidase	[57]
Chlorogenic acid (**18**)	Leaves and seeds	-	[25,26]
		Antimicrobial activity	[29]
		Antioxidant activity	[31]
		Anti-diabetic activity	[56]
		Hemolytic activity	[58]
		Neuroprotective effects	[59]
		Anti-obesity activity	[60]
		Antihepatotoxic effect	[61]
		Antibiofilm activity	[62]
Cinnamic acid (**19**)	Sprouts and seeds	-	[34]
*o*-Coumaric acid (**20**)	Leaves and seeds	-	[25,26]
		Allelopathic effect	[30]
		Antioxidant activity	[31]
*p*-Coumaric acid (**21**)	Leaves and seeds	-	[27,63]
		Antilisterial activity	[64]
*p*-Coumaric acid glucoside (**22**)	Seeds	-	[35]
8,5′-Diferulic acid (**23**)	Seeds	-	[25]
Ferulic acid (**24**)	Leaves, sprouts and seeds	-	[27,34,35]
		Antimicrobial activity	[29]
		Anti-apoptotic activity	[55]
		Antioxidant activity	[65]
		Cholesterol-lowering activity	[66]
		Anti-thrombosis and anti-atherosclerosis effects	[67,68]
		Anti-inflammatory activity	[69]
		Anti-cancer activity	[70]
Ferulic acid 4-*O*-glucoside (**25**)	Flour	-	[33]
Isoferulic acid (**26**)	Seeds	-	[35]
		Antioxidant activity	[71]
4’-Geranyloxyferulic acid (**27**)	Seeds	-	[72]
Rosmarinic acid (**28**)	Seeds	-	[25]
		Antimicrobial activity	[73]
		Anti-inflammatory activity	[74]
		Antioxidant activity	[75]
		Antimutagenicity activity	[76]
		Antiviral and anti-inflammatory effects	[77]
Sinapinic acid = *trans*-Sinapic acid (**29**)	Leaves	-	[27]
	Seeds	-	[25]
		Antioxidant activity	[44]
		Anxiolytic-like effects	[78]
		Cerebral protective and cognition-improving effects	[79]

**Table 3:** Flavones and their biological activities or functions.

Name	Quinoa Part Used for Isolation	Biological Activity or Function	Ref.
Acacetin (**30**)	Flour	-	[33]
		Antioxidant activity	[83]
		Spasmolytic and antinociceptive activities	[85]
		Antiproliferative activity	[86]
		Antiherpetic activity	[87]
		Anticancer activity	[88]
		Anti-inflammatory and antinociceptive activities	[89]
		Hypouricemic effect	[90]
Isovitexin (**31**)	Sprouts	-	[34]
		Anti-inflammatory and antioxidant activities	[84]
		Anti-neoplastic effect	[91]
		Anti-tumour activity	[92]
		Neuroprotective effect	[93]
		Anxiolytic property	[94]
		Anti-Alzheimer‘s disease	[95]
		Reduced postprandial blood glucose	[96]
		Inhibitory effect on α-glucosidase	[97]
		Inhibitory activity on rat lens aldose reductase	[98]
Orientin (**32**)	Seeds	-	[34]
		Anticancer activity	[7]
		Anti-inflammatory activity	[99]
		Antioxidant activity	[100]
		Antiapoptosis activity	[101]
		Antithrombotic and antiplatelet activities	[102]
		Antiproliferative activity	[103]
Vitexin (**33**)	Sprouts and seeds	-	[34]
		Anti-carcinogenic effect	[91]
		Anxiolytic property	[94]
		Anti-Alzheimer’s disease property	[95]
		Reduced postprandial blood glucose	[96]
		Inhibitory effect on α-glucosidase	[97]
		Induced apoptosis property	[104]
		Agonist-induced regulation of vascular contractility	[105]
		Antioxidant activity	[106]
		Anti-inflammatory activity	[107]
		Neuroprotective effect	[108]
		Anti-depressant effect	[109]
		Anti-convulsant effect	[110]
		Antiepileptic effect	[111]
		Anti-nociceptive effect	[112]
		Anti-hypoxia/ischemia injury	[113]
		Anti-ischemia/reperfusion injury	[114]
		Anti-thyroid effect	[115]
		Antimicrobial activity	[116]
		Anti-viral effect	[117]

**Table 4:** Flavonols and their biological activities or functions.

Name	Quinoa Part Used for Isolation	Biological Activity or Function	Ref.
Isorhamnetin (**34**)	Leaves	-	[27,125]
		Chemopreventive activity	[126]
		Antituberculosis activity	[127]
		Antioxidant activity	[128]
		Anti-tumor activity	[129,130]
		Inhibitory activity on farnesyl protein transferase	[131]
		Anti-inflammatory activity	[132]
		Anticoagulant activity	[133]
Kaempferol (**35**)	Leaves and seeds	-	[25,26,35]
		Antibacterial activity	[36]
		Antioxidant activitiy	[134]
		Inhibit UVB-induced COX-2 expression	[135]
		Anti-inflammatory activitiy	[136]
		Stimulate osteoblastic activity	[137]
Kaempferol 3-glucoside (**36**)	Seeds	-	[35]
Kaempferol 3-galactoside (**37**)	Seeds	-	[35]
Kaempferol 3-*O*-(2,6-di-α-l-rhamnopyranosyl)-β-d-galactopyranoside (**38**)	Seeds	Antioxidant activity	[49,120,138,139]
Kaempferol 3-*O*-β-d-apiofuranosyl-(1→2)-*O*-α-l-rhamnopyranosyl(1→6)-β-d-galactopyranoside (**39**)	Seeds	Antioxidant activity	[49,120,138]
Kaempferol 3-*O*-β-d-apiofuranosyl-(l→2)-β-d-galactopyranoside (**40**)	Seeds	Antioxidant activity	[120,138]
Kaempferol 3-*O*-α-l-rhamnopyranosyl-(1→2)-β-d-galactopyranoside (**41**)	Seeds	Antioxidant activity	[120]
Kaempferol 3-*O*-β-d-glucuronic acid (**42**)	Seeds	Antioxidant activity	[49]
Kaempferol 3,7-dirhamnoside (**43**)	Seeds	-	[35]
Morin (**44**)	Sprouts and seeds	-	[34]
		Anti-biofilm activity	[140]
		Anti-inflammatory activity	[141]
		Antitumor activity	[142]
		Inhibitory effect on the expression of α1 (I) collagen	[143]
		Antioxidant activity	[144]
		Anticancer activity	[145]
		Inhibited the increase of ROS and reduced the apoptotic cell	[146]
		Neuroprotective effect	[147]
		Hepatoprotective activity	[148]
Myricetin (**45**)	Seeds	-	[63]
		Antibacterial activity	[36]
		Antioxidant and prooxidant activities	[149]
		Anticancer activity	[150]
		Anti-inflammatory activity	[151]
		Analgesic activity	[152]
Quercetin (**46**)	Leaves and seeds	-	[27,35,125,139]
		COX-I and COX-II inhibition activity	[48]
		Stimulate osteoblastic activity	[137]
		Antioxidant and prooxidant activities	[149]
		Anti-inflammatory activity	[153]
		Cytotoxic activity	[154]
Quercetin 3-*O*-glucoside (**47**)	Flour	-	[33]
Quercetin-3-rutinoside (**48**)	Seeds	-	[35]
Quercetin 3-arabinoside (**49**)	Seeds	-	[35]
Quercetin 3-*O*-β-d-apiofuranosyl-(1→2)-α-l-rhamnopyranosyl-(1→6)-β-d-galactopyranoside (**50**)	Seeds	Antioxidant activity	[120,139]
Quercetin 3-*O*-(2,6-di-α-l-rhamnopyranosy)-β-d-galactopyranoside (**51**)	Seeds	Antioxidant activity	[49,120]
Quercetin 3-*O*-(2,6-di-*O*-α-rhamnopyranosyl)-β-glucopyranoside (**52**)	Seeds	-	[139]
Quercetin 3-*O*-β-d-apiofuranosyl-(1→2)-*O*-α-l-rhamnopyranosyl-(1→6)-β-d-galactopyranoside-3,4-dimethyl ether (**53**)	Seeds	Antioxidant activity	[49]
Rutin (**54**)	Leaves, sprouts and seeds	-	[27,34]
		Anti-diabetic activity	[56]
		Antioxidant activity	[155]
		Antiulcerogenic activity	[156]

**Table 5:** Flavanones and their biological activities.

Name	Quinoa Part Used for Isolation	Biological Activity or Function	Ref.
Hesperidin (**55**)	Seeds	-	[34]
		Neuroprotective effect	[147]
		Antioxidant and cytotoxic activities	[157]
		Anti-inflammatory activity	[158]
		Antifungal activity	[159]
		Anti-proliferative and apoptotic activities	[160]
		Protects the liver against drug-induced injury	[161]
		Cardioprotective activity	[162]
Neohesperidin (**56**)	Seeds	-	[34]
		Neuroprotective effect	[147]
		Antifungal activity	[159]
		Antioxidant activity	[163]
		Induces cell apoptosis	[164]
Naringin (**57**)	Seeds	-	[35]
		Antifungal activity	[159]
		Antioxidative activity	[165]
		Anti-osteoporosis activity	[166]
		Anti-inflammatory activity	[167]

**Table 6:** Flavanols and their biological activities or functions.

Name	Quinoa Part Used for Isolation	Biological Activity or Function	Ref.
Catechin (**58**)	Seeds	-	[25]
		Antioxidant activity	[149]
		Antimutagenic activity	[169]
		Anti-metastatic activity	[170]
		Antifungal activity	[171]
		Apoptosis-inducing activity	[172]
Epicatechin (**59**)	Seeds	-	[35]
		Antimutagenic activity	[169]
		Antioxidant activity	[173]
		Antiproliferative activity	[174]
Epigallocatechin (**60**)	Seeds	-	[33,35]
		Antioxidant activity	[168]

**Table 7:** Isoflavones and their biological activities or functions.

Name	Quinoa Part Used for Isolation	Biological Activity or Function	Ref.
Biochanin A (**61**)	Seeds	-	[35]
Daidzein (**62**)	Seeds	-	[176]
		Antioxidant activity	[177]
		Enhance adipocyte differentiation and PPARγ transcriptional activities	[178]
		Affected human nonhormone-dependent cervical cancer cells	[179]
		Modulate in vitro rat uterine contractile activity	[180]
		Anti-hypoxia activity	[181]
		Antithrombotic and antiallergic activities	[182]
		Chemoprotective activity	[183]
		Inhibits bone loss in ovariectomized mice	[184]
		Antiproliferative activity	[185,186]
Genistein (**63**)	Seeds	-	[176]
		Antiproliferative activity on human breat cancer cells	[185,186]
		Modulate in vitro rat uterine contractile activity	[180]
		Antioxidant activity	[187]
		Inhibitory activity on tyrosine-specific protein kinases	[188]
		Antitumor activity	[189]
		Cytotoxic activity and anticancer activities	[190]
		Antitumor and antiangiogenic activities	[191,192]
		Antibacterial activity	[193]
		Inhibition of cyclooxygenase-2 activity	[194]
		Antiprostate cancer activity	[195]
		Antileukemic activity	[196]
		Induction of quinone reductase activity	[197]
		Induces growth arrest and suppresses telomerase activities	[198]
Prunetin (**64**)	Seeds	-	[25]
		Anti-inflammatory activity	[199]
Puerarin (**65**)	Seeds	-	[35]
		Antithrombotic and antiallergic activities	[182]
		Anti-apoptosis activity	[200]
		Antioxidant activity	[201]
		Antihyperglycemic effect	[202]

**Table 8:** Monoterpenoids and their biological activities or functions.

Name	Quinoa Part Used for Isolation	Biological Activity or Function	Ref.
*cis*-Ascaridole (**66**)	Leaves	-	[204]
*cis*-Isoascaridole (**67**)	Leaves	-	[204]
Camphene (**68**)	Leaves	-	[204]
Camphor (**69**)	Leaves	-	[204]
*trans*-Carveol (**70**)	Leaves	-	[204]
*p*-Cymene (**71**)	Leaves	-	[204]
*p*-Mentha-1(7),8-diene (**72**)	Leaves	-	[204]
*trans*-*p*-Menth-2-en-1-ol (**73**)	Leaves	-	[204]
Penstebioside (**74**)	Flour	-	[33]
β-Pinene (**75**)	Leaves	-	[204]
Pinocarvone (**76**)	Leaves	-	[204]
α-Terpinene (**77**)	Leaves	-	[204]
γ-Terpinene (**78**)	Leaves	-	[204]
		Antibacterial activity	[205]
Terpin-1-ol (**79**)	Leaves	-	[204]
α-Terpinyl acetate (**80**)	Leaves	-	[204]

**Table 9:** Oleanolic acid derivatives and their biological activities or functions.

Name	Quinoa Part Used for Isolation	Biological Activity or Function	Ref.
Oleanolic acid (**82**)	Seeds and bran	-	[217,233]
		Antimicrobial activity	[220,221]
		Anti-HIV activity	[222]
		Anti-inflammatory activity	[223]
		Antioxidant activity	[225]
		Antifertility activity	[226]
		Antitumor activity	[227,228]
		Inhibitory activities on serin protease and porcine pancreatic elastase	[232]
Methyl oleanate (**90**)	Bran	Anti-inflammatory activity	[234]
3-*O*-α-l-Arabinopyranosyl-(1→3)-β-d-glucuronopyranosyl oleanolic acid 28-*O*-β-d-glucopyranosyl ester (**91**)	Seeds	-	[235,236]
3-*O*-β-d-Glucopyranosyl oleanolic acid (**92**)	Seeds	-	[237]
		Antidiabetogenic activity	[230]
		Anti-inflammatory activity	[224]
		Hemolytic activity	[231]
3-*O*-β-d-Glucopyranosyl-(1→3)-α-l-arabinopyranosyl oleanolic acid 28-*O*-β-d-glucopyranosyl ester (**93**)	Flowers, fruits, seeds and bran	-	[11,235,236]
3-*O*-β-d-Glucopyranosyl-(1→2)-β-d-glucopyranosyl-(1→3)-α-l-arabinopyranosyl oleanolic acid 28-*O*-β-d-glucopyranosyl ester (**94**)	Flowers, fruits, seeds and bran	-	[11,217,238]
3-*O*-β-d-Glucuropyranosyl oleanolic acid (**95**)	Seeds	-	[208,238]
3-*O*-β-d-Glucuronopyranosyl oleanolic acid 28-*O*-β-d-glucopyranosyl ester (**96**)	Flowers, fruits, seeds and bran	Hemolytic activity	[11,208,217,236]
3-*O*-β-d-Xylopyranosy-(1→3)-β-d-glucuronopyranosyl oleanolic acid (**97**)	Seeds	-	[237]
3-*O*-β-d-Xylopyranosy(1→3)-6-methyl-β-d-glucuronopyranosyl oleanolic acid (**98**)	Seeds	-	[237]
3-*O*-β-d-Xylopyranosyl-(1→3)-β-d-glucuronopyranosyl oleanolic acid 28-*O*-β-d-glucopyranosyl ester (**99**)	Flowers, fruits, seeds and bran	-	[11,217,237]

**Table 10:** Hederagenin derivatives and their biological activities or functions.

Name	Quinoa Part Used for Isolation	Biological Activity or Function	Ref.
Hederagenin (**83**)	Seeds and bran	-	[215,216,217]
		Inhibitory activity on serin protease, and porcine pancreatic elastase	[232]
		Cytotoxic activity on P-388 mouse lymphoma, L-1210 mouse lymphomatic leukemia, HL-60 human promyelocytic leukemia and SNU-5 human stomach cancer cells	[248,249]
		Haemolytic activity	[250]
		Anti-inflammatory activity	[244]
		Antidermatophytic activity	[251]
		Antitrichomonas activity	[252]
		Inducing apoptosis in human LoVo colon cells	[247]
3-*O*-α-l-Arabinopyranosyl hederagenin (**100**)	Seeds	-	[208]
		Molluscicidal activity	[140,253]
		Cytotoxic activity on human carcinoma and melanoma cell lines DLD-1, PA1, A549, MCF7, PC3, and M4	[241]
		Antifungal activity	[242]
		Leishmanicidic activity	[243]
		Antidermatophytic activity	[251]
3-*O*-α-l-Arabinopyranosyl hederagenin 28-*O*-β-d-glucopyranosyl ester (**101**)	Flowers, fruits, seeds and bran	-	[11,208,216,217]
		Antidermatophytic activity	[251]
		Anticomplementary activity	[254]
3-*O*-β-d-Glucopyranosyl-(1→3)-α-l-arabinopyranosyl hederagenin (**102**)	Seeds and bran	-	[208,216,238]
		Cytotoxic activity on A549, SK-OV-3, SK-MEL-2, XF498 and HCT15	[255]
3-*O*-β-d-Glucopyranosyl-(1→3)-β-d-galactopyranosyl hederagenin (**103**)	Bran	-	[216]
3-*O*-β-d-Glucopyranosyl-(1→3)-α-l-arabinopyranoside hederagenin 28-*O*-β-d-glucopyranosyl ester (**104**)	Flowers, fruits, seeds and bran	-	[11,208,216,217,236,238,256]
3-*O*-β-d-Glucopyranosyl-(1→3)-β-d-galactopyranosyl hederagenin 28-*O*-β-d-glucopyranosyl ester (**105**)	Flowers, fruits, seeds and bran	-	[11,216,238]
3-*O*-β-d-Glucopyranosyl-(1→4)-β-d-glucopyranosyl-(1→4)-β-d-glucopyranosyl hederagenin 28-*O*-β-d-glucopyranosyl ester (**106**)	Seeds	-	[256]
3,23-Bis(*O*-β-d-glucopyranosyloxy) olean-12-en-28-oic acid 28-*O*-β-d-glucopyranosyl-(1→3)-α-l-arabinopyanosyl ester (**107**)	Seeds	-	[257]
3-*O*-β-d-Glucuronopyranosyl hederagenin 28-*O*-β-glucopyranosyl ester (**108**)	Flowers, fruits, seeds and bran	-	[11,217,238]
3-*O*-β-d-Xylopyranosyl-(1→3)-β-d-glucuronopyranosyl hederagenin 28-*O*-β-d-glucopyranosyl ester (**109**)	Bran	-	[217]

**Table 11:** Spergulagenic acid derivatives and their biological activities or functions.

Name	Quinoa Part Used for Isolation	Biological Activity or Function	Ref.
3-*O*-α-l-Arabinopyranosyl-(1→3)-β-d-glucuronopyranosyl spergulagenic acid 28-*O*-β-d-glucopyranosyl ester (**110**)	Seeds	-	[256]
3-*O*-β-d-Glucopyranosyl-(1→2)-β-d-glucopyranosyl-(1→3)-α-l-arabinopyranosyl spergulagenic acid (**111**)	Bran	-	[217]
3-*O*-β-d-Glucopyranosyl-(1→2)-β-d-glucopyranosyl-(1→3)-α-l-arabinopyranosyl spergulagenic acid 28-*O*-β-d-glucopyranosyl ester (**112**)	Seeds	-	[256]

**Table 12:** Serjanic acid derivatives and their biological activities or functions.

Name	Quinoa Part Used for Isolation	Biological Activity or Function	Ref.
Serjanic acid (**85**)	Flowers, fruits, seeds and bran	Cytotoxic activity on HeLa cell line	[11,217]
3-*O*-α-l-Arabinopyranosyl serjanic acid 28-*O*-β-d-glucopyranosyl ester (**113**)	Flowers, fruits, seeds and bran	Cytotoxic activity on Hela cell line	[11]
3-*O*-β-d-Glucopyranosyl-(1→3)-α-l-arabinopyranosyl serjanic acid 28-*O*-β-d-glucopyranosyl ester = 3-*O*-β-d-Glucopyranosyl-(1→3)-α-l-arabinopyranosyl-30-*O*-methyl spergulagenate 28-*O*-β-d-glucopyranosyl ester (**114**)	Flowers, fruits, seeds and bran	-	[11,236,238]
3-*O*-β-d-Glucopyranosyl-(1→2)-β-d-glucopyranosyl-(1→3)-α-l-arabinopyranosyl serjanic acid 28-*O*-β-d-glucopyranosyl ester (?) = 3-*O*-β-d-Glucopyranosyl-(1→2)-β-d-glucopyranosyl-(1→3)-α-l-arabinopyranosyl-30-*O*-methyl spergulagenate 28-*O*-β-d-glucopyranosyl ester (**115**)	Flowers, fruits, seeds and bran	-	[11,217,238,256]
3-*O*-β-d-Glucuronopyranosyl serjanic acid 28-*O*-β-d-glucopyranosyl ester (**116**)	Flowers, fruits, seeds and bran	Cytotoxic activity on HeLa cell line	[11]

**Table 13:** Phytolaccagenic acid derivatives and their biological activities or functions.

Name	Quinoa Part Used for Isolation	Biological Activity or Function	Ref.
Phytolaccagenic acid (**86**)	Bran	-	[216,217]
	Bran	Anti-inflammatory activity	[234]
3-*O*-α-l-Arabinopyranosyl phytolaccagenic acid 28-*O*-β-d-glucopyranosyl ester (**117**)	Flowers, fruits, seeds and bran	-	[11,208,216,217,238]
3-*O*-α-l-Arabinopyranosyl-(1→3)-β-d-glucuronopyranosyl phytolaccagenic acid 28-*O*-β-d-glucopyranosyl ester (**118**)	Seeds	-	[235,236,256]
3-*O*-β-d-Galactopyranosyl-(1→3)-β-d-glucopyranosyl phytolaccagenic acid 28-*O*-β-d-glucopyranosyl ester (**119**)	Seeds	-	[238]
3-*O*-β-d-Glucopyranosyl-(1→3)-α-l-arabinopyranosyl phytolaccagenic acid (**120**)	Seeds	Antifungal activity	[208,238]
3-*O*-β-d-Glucopyranosyl-(1→3)-α-l-arabinopyranosyl phytolaccagenic acid 28-*O*-β-d-glucopyranosyl ester (**121**)	Flowers, fruits, seeds and bran	-	[11,208,216,217,235,236,238]
3-*O*-β-d-Glucopyranosyl-(1→3)-β-d-galactopyranoside phytolaccagenic acid 28-*O*-β-d-glucopyranosyl ester (**122**)	Flowers, fruits, seeds and bran	-	[11,216,217,238]
3-*O*-β-d-Glucopyranosyl(1→2)-β-d-glucopyranosyl-(1→3)-α-l-arabinopyranoside phytolaccagenic acid 28-*O*-β-d-glucopyranosyl ester (**123**)	Flowers, fruits, seeds and bran	-	[11,217,235,238]
3-*O*-β-d-Glucopyranosyl-(1→4)-β-d-glucopyranosyl-(1→4)-β-d-glucopyranosyl phytolaccagenic acid 28-*O*-β-d-glucopyranosyl ester (**124**)	Flowers, fruits, seeds and bran	-	[11,256]
3-*O*-β-d-Glucopyranosyl-(1→3)-β-d-xylopyranosyl-(1→2)-β-d-glucopyranosyl phytolaccagenic acid 28-*O*-β-d-glucopyranosyl ester (**125**)	Seeds	-	[235]

**Table 14:** Gypsogenin derivatives and their biological activities or functions.

Name	Quinoa Part Used for Isolation	Biological Activity or Function	Ref.
Gypsogenin = 3β-Hydroxy-23-oxo-olean-12-en-28-oic acid (**87**)	Flowers, fruits, seeds and bran	Cytotoxic activity on Hela cell line	[11]
3-*O*-β-d-Glucopyranosyl-(1→3)-α-l-arabinopyranosyl 23-oxo-olean-12-en-28-oic acid 28-*O*-β-d-glucopyranosyl ester = 3β-[(*O*-β-d-Glucopyranosyl-(1→3)-α-l-arabinopyranosyl)oxy]-23-oxo-olean-12-en-28-oic acid 28-*O*-β-d-glucopyranosyl ester (**126**)	Flowers, fruits, seeds and bran	Cytotoxic activity on HeLa cell line	[11]

**Table 15:** 3β-Hydroxy-27-oxo-olean-12-en-28-oic acid derivatives and their biological activities or functions.

Name	Quinoa Part Used for Isolation	Biological Activity or Function	Ref.
3β-Hydroxy-27-oxo-olean-12-en-28-oic acid (**88**)	Flowers, fruits, seeds and bran	Cytotoxic activity on HeLa cell line	[11]
3-*O*-β-d-Glucopyranosyl-(1→3)-α-l-arabinopyranosyl 27-oxo-olean-12-en-28-oic acid 28-*O*-β-d-glucopyranosyl ester = 3β-[(*O*-β-d-Glucopyranosyl-(1→3)-α-l-arabinopyranosyl)oxy]-27-oxo-olean-12-en-28-oic acid 28-*O*-β-d-glucopyranoside (**127**)	Flowers, fruits, seeds and bran	Cytotoxic activity on Hela cell line	[11]

**Table 16:** 3β,23,30-Trihydroxy-olean-12-en-28-oic acid triterpenoids and their biological activities or functions.

Name	Quinoa Part Used for Isolation	Biological Activity or Function	Ref.
3β,23,30-Trihydroxy-olean-12-en-28-oic acid (**89**)	Flowers, fruits, seeds and bran	-	[11]
3-*O*-β-d-Glucopyranosyl-(1→3)-α-l-arabinopyranosyl 3β,23,30-trihydroxyolean-12-en-28-oic acid 28-*O*-β-d-glucopyranosyl ester (**128**)	Flowers, fruits, seeds and bran	-	[11,214]

**Table 17:** Other triterpenoids and their biological activities or functions.

Name	Quinoa Part Used for Isolation	Biological Activity or Function	Ref.
**Tetracyclic triterpenoids**			
Citrostadienol (**129**)	Seeds	-	[261]
		Anticomplementary activity	[262]
Gramisterol (**130**)	Seeds	-	[261]
		Anti-cancer activity on mouse leukemic cell line WEHI-3	[8]
24-Methylene-cycloartenol (**131**)	Seeds	-	[261]
Parkeol (**132**)	Seeds	-	[261]
			
**Pentacyclic triterpenoids**			
α-Amyrin (**133**)	Seeds	-	[215]
		Antibacterial activity	[263]
		Antidiabetic effect	[264]
		Antioxidant activity	[265]
		Inhibitory activity against human oxidosqualene cyclase	[266]
β-Amyrin (**134**)	Seeds	-	[215]
		Antibacterial activity	[263]
		Antioxidant activity	[265]
		Inhibitory activity against human oxidosqualene cyclase	[266]
		Antifeedant and growth regulating activities	[267]
		Insecticidal activity	[268]
Echinocystic acid (**135**)	Seeds	-	[215]
Erythrodiol (**136**)	Seeds	-	[215,261]
		Antibacterial activity	[269]
		Melanogenesis-inhibitory activity	[270]
		Protecting the cardiovascular system	[271]
		Antiproliferative and apoptotic activity	[272]
3β,23-Dihydroxy-olean-12-ene-28,30-dioic acid (**137**)	Seeds	-	[208]
2β,3β,23-Trihydroxy-olean-12-ene-28,30-dioic acid 30-methyl ester (**138**)	Bran	-	[216]
Queretaroic acid (**139**)	Seeds	-	[215]
Ursolic acid (**140**)	Seeds	-	[215]
		Spasmolytic and antinociceptive activities	[85]
		Cytotoxic activity	[270]
		Anticancer activity	[273,274]

**Table 18:** Meroterpenoids and their biological activities or functions.

Name	Quinoa Part Used for Isolation	Biological Activity or Function	Ref.
α-Tocopherol (**141**)	Seeds	-	[283]
		Antioxidative, antihypercholesterolemic, anticancer, neuroprotective activities	[284]
β-Tocopherol (**142**)	Seeds	-	[37]
		Antioxidative, antihypercholesterolemic, anticancer, neuroprotective activities	[284]
γ-Tocopherol (**143**)	Seeds	-	[283]
		Antioxidative, antihypercholesterolemic, anticancer, neuroprotective activities	[284]
δ-Tocopherol (**144**)	Seeds	-	[37]
		Antioxidative, antihypercholesterolemic, anticancer, neuroprotective activities	[284]
α-Tocotrienol (**145**)	Seeds	-	[275]
		Antioxidant and anti-inflammatory activities	[282]
β-Tocotrienol (**146**)	Seeds	-	[275]
		Antioxidant and anti-inflammatory activities	[282]

**Table 19:** C_27_-Steroids and their biological activities or functions.

Name	Quinoa Part Used for Isolation	Biological Activity or Function	Ref.
Cholesterol (**147**)	Seeds	-	[285,288]
20-Hydroxyecdysone (**148**)	Seeds	Antioxidant activity	[289]
		Inhibitory activity on collagenase	[287]
		Insecticidal activity	[290]
20,26-Dihydroxyecdysone (**149**)	Seeds	Antioxidant activity	[289]
		Inhibitory activity on collagenase	[287]
2-Deoxy-20-hydroxyecdysone (**150**)	Seeds	-	[22]
3-*epi*-2-Deoxy-20-hydroxyecdysone (**151**)	Seeds	-	[22]
2-Deoxy-20,26-dihydroxyecdysone (**152**)	Seeds	-	[22]
20-Hydroxyecdysone 22-glycolate (**153**)	Seeds	Antioxidant activity and inhibitory activity on collagenase	[287]
24,25-Dehydroinokosterone (**154**)	Seeds	-	[22]
25,27-Dehydroinokosterone (**155**)	Seeds	-	[22]
Lathosterol (**156**)	Seeds	-	[261]
Polypodine B (**157**)	Seeds	-	[22]

**Table 20:** C_28_-Steroids and their biological activities or functions.

Name	Quinoa Part Used for Isolation	Biological Activity or Function	Ref.
Brassicasterol (**158**)	Seeds	-	[292]
Campestanol (**159**)	Seeds	-	[261]
Campesterol = Δ^5^-Campesterol (**160**)	Seeds	-	[261,285,288]
		Antiangiogenic activity	[291]
Δ^7^-Campesterol (**161**)	Seeds	-	[285,288]
Dacrysterone (**162**)	Seeds	-	[22]
Episterol (**163**)	Seeds	-	[261]
Ergost-7-en-3β-ol = Δ^7^-Ergostenol (**164**)	Seeds	-	[261]
Kancollosterone (**165**)	Seeds	-	[293]
Makisterone A (**166**)	Seeds	Antioxidant activity	[289]
		Inhibitory activity on collagenase	[287]
24-*epi*-Makisterone A (**167**)	Seeds	Antioxidant activity	[289]
		Inhibitory activity on collagenase	[287]
24(28)-Dehydromakisterone A (**168**)	Seeds	Antioxidant activity	[289]
		Inhibitory activity on collagenase	[287]
26-Hydroxy-24(28)-dehydromakisterone A (**169**)	Seeds	Antioxidant activity, inhibitory activity on collagenase	[287]
5β-Hydroxy-24(28)-dehydromakisterone A (**170**)	Seeds	-	[22]
24-Methyl-20,26-dihydroxyecdysone (**171**)	Seeds	-	[22]
	Seeds	Antioxidant activity	[287]

**Table 21:** C_29_-Steroids and their biological activities or functions.

Name	Quinoa Part Used for Isolation	Biological Activity or Function	Ref.
Δ^5^-Avenasterol = Δ^5,24(28)^-Avenasterol (**172**)	Seeds	-	[261,285,288]
Δ^7^-Avenasterol = Δ^7,24(28)^-Avenasterol (**173**)	Seeds	-	[261]
Makisterone C (**174**)	Seeds	-	[22]
Sitostanol (**175**)	Seeds	-	[261]
*β*-Sitosterol (**176**)	Seeds	-	[285,288]
		Insecticidal activity	[268]
		Anti-inflammatory activity	[294]
		Anti-oxidant activity	[295]
		Antidiabetic activity	[296]
		Inducing apoptosis	[302]
		Hypocholesterolemic activity	[303,304]
		Angiogenic effect	[305]
		Genotoxicity effect	[306]
		Anthelminthic and Anti-mutagenic activity	[307]
		Immunomodulatory activity	[308]
		Neuroprotection effect	[309]
Stigmast-4-en-3-one (**177**)	Seeds		[292]
Stigmast-4,22-dien-3-one (**178**)	Seeds		[292]
Stigmast-8-en-3-ol (**179**)	Seeds	-	[261]
Δ^7^-stigmastenol (**180**)	Seeds	-	[261]
Stigmasterol = Δ^5^-Stigmasterol (**181**)	Seeds	-	[261]
		Anti-inflammatory activity	[297]
		Anti-tumor activity	[298]
		Antifungal activity	[299]
		Anti-hypercholestrolemic activity	[300]
		Cytotoxicity activity	[301]
		Anti-osteoarthritic activity	[310]
Δ^7^-Stigmasterol (**182**)	Seeds	-	[285,288]
	Seeds	-	[261]

**Table 22:** Nitrogen-containing metabolites and their biological activities or functions.

Name	Quinoa Part Used for Isolation	Biological Activity or Function	Ref.
Amaranthin (**183**)	Seeds	-	[316]
Betanin (**184**)	Seeds	-	[35]
		Antioxidant activity	[317]
Isobetanin (**185**)	Seeds	-	[35]
Betaine (**186**)	Seeds	-	[35]
3-Carboxy-1-(2-sulfoethyl)-pyridinium (**187**)	Seeds	-	[313]
Dopaxanthin (**188**)	Seeds	-	[316]
		Antioxidant activity	[318]
Indicaxanthin (**189**)	Seeds	-	[316]
Miraxanthin V (**190**)	Seeds	-	[316]
Trigonelline (**191**)	Seeds	-	[313]
		Anti-invasive activity	[319]
		Hypoglycemic effect	[320]
Trigonelline glucosylester (**192**)	Seeds	-	[313]
Trigonelline methylester (**193**)	Seeds	-	[313]
